# Are Primary Outcomes Really Primary? An Exploratory Cross-Sectional Nationwide Web-Based Survey Study for Outcomes Reflecting Real Symptoms and Needs of Patients with Lumbar Disc Herniation

**DOI:** 10.3390/healthcare11182598

**Published:** 2023-09-21

**Authors:** Doori Kim, Soo-Jin Kim, Yoon Jae Lee, Chang Sop Yang, Chang-Hyun Han, In-Hyuk Ha

**Affiliations:** 1 Jaseng Spine and Joint Research Institute, Jaseng Medical Foundation, 540 Gangnam-daero, Gangnam-gu, Seoul 06110, Republic of Korea; doori.k07@gmail.com (D.K.); ekffurkfl@gmail.com (S.-J.K.); goodsmile8119@gmail.com (Y.J.L.); 2 Clinical Medicine Division, Korea Institute of Oriental Medicine, 1672 Yuseong-daero, Yuseong-gu, Daejeon 34054, Republic of Korea; yangunja@kiom.re.kr; 3 Korean Convergence Medical Science, School of Korea Institute of Oriental Medicine, University of Science & Technology (UST), 1672 Yuseong-daero, Yuseong-gu, Daejeon 34054, Republic of Korea; 4 KM Science Research Division, Korea Institute of Oriental Medicine, 1672 Yuseong-daero, Yuseong-gu, Daejeon 34054, Republic of Korea

**Keywords:** lumbar disc herniation, primary outcomes, clinical trials, treatment efficacy, stable treatment effect, safety, functional recovery, questionnaire study

## Abstract

As primary outcomes differ among clinical lumbar disc herniation (LDH) studies, this study aimed to explore outcomes reflecting real-world patient experiences through an exploratory questionnaire survey. Those diagnosed with LDH having radiating leg pain in South Korea in November of 2022 (N = 500) were administered a questionnaire including basic characteristics, disease onset, symptoms and severity, priority symptoms for improvement, and important treatment factors. Outcome measures included the identification of priority symptoms and disabilities. Most common symptoms were numbness in the leg (N = 435, 87.0%) and back pain (N = 406, 81.2%); most common disabilities were discomfort in sitting (N = 323, 64.6%) and lifting (N = 318, 63.6%). The highest priority symptom was back pain (N = 242, 48.4%). A satisfactory degree of symptom improvement was a decrease of at least 3 points on the numeric rating scale. The majority of respondents preferred improvement in disability over pain (N = 270, 55.8%), a stable effect over a rapid effect (N = 391, 78.2%), and safety over treatment efficacy (N = 282, 56.4%). Safety (N = 129, 25.8%) and cost (N = 111, 22.2%) were the most important treatment factors. Improvements in back pain, leg pain, sitting, and sleeping were prioritized, and safety, stable treatment effect, and functional recovery were desired. Clinical trials for LDH should be designed to reflect this real-world patient need. Further study to examine the patients’ symptoms and needs in details is needed.

## 1. Introduction

Low back pain (LBP) and radiating leg pain are highly prevalent worldwide, with the lifetime prevalence of LBP being as high as 66% [1]. Moreover, the World Health Organization reports that up to 1 in 7 people experience back pain [2]. LBP is associated with high socioeconomic costs and is the leading cause of years lived with disability [3]. In the United States, the annual cost of back pain management is 200 billion USD [4].

The causes of back pain are myriad, the most common of which is lumbar disc herniation (LDH) [5]. In LDH, tears and fissures in the annulus fibrosus of the disc lead to herniation of the disc material, and the extruded disc material contacts the thecal sac or lumbar nerve roots, possibly exerting pressure. Consequently, localized pressure and neurochemical inflammatory factors lead to a combination of nerve root ischemia and inflammation [6]. Depending on the specific nerve root affected, symptoms may include back pain, radiculopathy in the leg, paresthesia, or weakness [7]. Although LDH is not life-threatening, it may become chronic in some cases, in which patients experience persistent and recurrent symptoms over their lifetime [8].

Accordingly, many clinical trials on LDH have been conducted or are underway. An important aspect of designing clinical trials is the selection of outcomes in the target population to whom the results will be applied. Particularly, in clinical studies evaluating the effectiveness of specific treatments, the priority outcomes that reflect real-world symptoms and conditions are instrumental.

The numeric rating scale (NRS) scores for radiating leg pain, Oswestry disability index (ODI) score, and Roland–Morris disability questionnaire (RMDQ) score are commonly used as primary outcomes. The NRS is a numeric pain scale for the objective representation of subjective pain felt by individual patients (0 representing no pain and 10 representing the worst pain and discomfort imaginable) [9]. The ODI is a 10-item questionnaire developed for quantified assessment of functional impairment for LBP. Each item is divided into 6 levels and assigned 0–5 points, respectively. The higher the score, the more severe the disability [10,11]. The RMDQ is also one of the most common instruments used to assess the functional status of patients with LBP [12]. It is derived from the Sickness Impact Profile that consists of 24 items reflecting a variety of daily living activities; each item is scored 1 if declared applicable to the respondent and 0 if not, and thus, the total score can vary from 0 (no disability) to 24 (severe disability) [13,14]. However, primary outcomes differ between studies. Among studies on sciatica or LDH, some use the NRS score of radiating leg pain and ODI score as the primary outcome, while others assign the physical component score of the Short-Form and ODI score [15]; RMDQ score, intensity of radiating leg pain, and global perceived recovery [16]; or visual analog scale score, ODI score, and pressure pain threshold for leg and back pain as primary outcomes [17].

Despite these differences, no study has yet discussed the rationale for selecting these primary outcomes or their priority over other outcomes. In addition, the effects of treatment cannot be fully elucidated with a limited number of outcomes. Therefore, this questionnaire survey study aimed to explore the most appropriate outcomes to reflect the real needs and concerns of patients with LDH and provide guidance for outcomes in clinical trials.

## 2. Materials and Methods

### 2.1. Study Design

This cross-sectional nationwide web-based survey study was conducted using Opensurvey, a Korean mobile application survey company. Opensurvey selected respondents who met the inclusion criteria through a screening survey, stratified the respondents by age and sex, and distributed survey links. A total of 500 respondents were selected from 100 participants per age group, and the survey link was closed after the selection was completed. Survey data were collected between 15 and 16 November 2022.

Before commencing the survey, all respondents were asked to indicate their willingness to participate by checking an online consent form that included a brief introduction to the study, inclusion/exclusion criteria, and a confidentiality clause. Only the patients who agreed to participate were administered the survey. The survey was conducted anonymously, and personally identifiable information was encrypted and provided only to the investigators.

### 2.2. Respondents

The inclusion criteria were as follows: diagnosed with LDH by clinicians through clinical symptoms, physical examination, and radiological assessment; age of 19–69 years; presence of LDH-related symptoms such as pain, numbness, weakness, or tingling in the legs; and agreement to sign the informed consent form. Although magnetic resonance imaging (MRI) is considered the most appropriate test to confirm the presence of LDH [18], LDH can also be diagnosed through clinical symptoms, physical examination, and other radiological assessment [19]. Therefore, for this study, we included patients who had radiculopathy, which is a representative symptom of LDH and were diagnosed with LDH by clinicians through various evaluations.

It was judged that 100 people per age group was the minimum number to produce meaningful statistics; thus, a total of 500 respondents (100 patients from each of the 20s, 30s, 40s, 50s, and 60s age groups) were recruited using age-stratified sampling with nationwide panels. It was also determined by referring previous study that of 500 was “very good” for a survey study [20]. Meanwhile, patients in their 70s or older were excluded as disc patients are relatively young, as can be seen from studies showing that the average age of LDH patients is in their 40s [21] and that LDH incidence decreases with age in those over 60 years of age [22].

This study was conducted in accordance with the Declaration of Helsinki and approved by the Institutional Review Board of Jaseng Hospital of Korean Medicine (approval no.: JASENG 2022-10-027; approval date: 31 December 2022). Written patient consent forms were waived owing to the study design as a mobile application survey, and all participants expressed their consent by checking the consent form provided online. If a patient declined participation or withdrew consent, the collection of personal information was immediately discontinued, and the information discarded.

### 2.3. Survey Questionnaires

The questionnaire was developed based on previous studies [10,11,23] and interviews [24] conducted by the research team. Interviews were conducted along with published interview studies, and patients complained most often of back pain and various symptoms in the legs. In addition, the keywords of livelihood, safety, and recurrence appeared often. These common keywords were then used to development of the questionnaire. The questionnaire consisted of 52 questions in five parts: basic characteristics, onset, symptom and severity, symptoms requiring priority in improvement, and important factors of treatment. The survey could be completed in approximately 10 min.

Basic characteristics included sex, age, area of residence, and occupation. Age was categorized into the 20s (including age 19 years), 30s, 40s, 50s, and ≥60s groups. The area of residence was classified into Seoul, the capital area, metropolitan cities, and other areas. Occupations were classified according to the Korean Standard Classification of Occupations guidelines as follows: managers; professionals; clerks; service workers; sales workers; skilled agricultural, forestry, and fishery workers; craft and related trade workers; plant and machine operators and assemblers; elementary occupations; armed forces occupations; and people without formal occupation (including housewives and students).

### 2.4. Onset

The first onset of symptoms and recent onset including recurrence and exacerbation were investigated. The onset was selected from the following ranges: ≤4 weeks, 4–12 weeks, 12 weeks to 12 months, and ≥12 months. In addition, the time of LDH diagnosis and whether the respondent underwent MRI scans at the time of diagnosis were also determined. The specific time point and event that triggered the symptoms were investigated by asking the respondents to provide a written description.

### 2.5. Symptom and Severity

LDH symptoms were classified into symptoms and disabilities. Symptoms were classified as back pain (excluding pelvic and hip pain), pelvic pain (including hip pain), radiating leg pain, numbness in the leg, weakness in the leg, and tingling in the leg (feeling as if the skin of the affected area was someone else’s skin or having a dull sensation) for a more objective assessment. In addition, the severity of each symptom was evaluated using the NRS [9,25], a numeric scale for objective representation of the subjective intensity of symptoms experienced by the patient. The respondents rated their pain from 0 to 10, with 0 being no pain and 10 being the most severe.

Meanwhile, the types of disability included were classified as discomfort in personal care (dressing, washing), lifting, walking, sitting, standing, sleeping, and traveling (including driving); impacting social life (social activities with people); anxiety/depression; productivity loss; and gastrointestinal problems (from medications for LDH symptoms). The severity of each disability was also assessed using categorical options based on the ODI. The disability severity questionnaire is presented in Supplementary File S1.

### 2.6. Priority Symptoms for Improvement

The respondents prioritized symptoms for improvement by selecting one symptom each for the following questions “What is the symptom you would like to improve the most?” and “What is the symptom you would like to improve the second most?” For each selected symptom, the respondents then answered the following question using numbers from 0 to 10: “What NRS score would you consider for a satisfactory level of improvement for the selected symptom?” In the instance that respondents listed only one symptom, they did not respond to the question about the second symptom. Similarly, the first and second disabilities that the respondent aspired to improve the most were selected. The satisfactory level of improvement for the selected type of disability was classified based on answers of improvement of 100% (no residual symptoms), 80% or more, 50% or more, 30% or more, and 20% or more.

### 2.7. Important Factors in Treatment

Participant opinion on important factors of treatment was assessed by asking the participants to select between reduced pain and improvement in functional disability, between rapid effect and stable/lasting effect even if the improvement was slow, and between treatment effect and safety. In addition, patients were asked to choose three items in order of importance from appropriate cost, non-painful and comfortable treatment, rapid effect, overall condition, safety, time taken for treatment, and duration of improvement.

### 2.8. Statistical Analysis

A frequency analysis was performed for each respondent characteristic for the survey responses, and a two-tailed test was used to perform the statistical analysis, with the significance level set at 5%. As for basic characteristics, categorical variables are expressed as the frequency and percentage (N, %), while continuous variables are expressed as the mean and standard deviation.

The presence or absence of each symptom caused by LDH is reported as the frequency and percentage (N, %), and the differences in the rate of each symptom according to sex, age, symptom duration, and severity of symptoms were tested using the chi-square test.

Regarding disability, the status of presence or absence is reported as frequency and percentage (N, %), and the difference in the incidence of each disability according to sex and age was analyzed using a chi-square test or Monte Carlo simulation method [26,27].

The types of symptoms and disability requiring priority in improvement are reported as the frequency and percentage (N, %). The frequency and percentage were calculated for responses including only the first priority and both the first/second priorities. In addition, the percentage of patients with each symptom and disability was calculated. Differences by respondent characteristics were tested using the chi-square test. For symptoms, differences were tested according to sex, age, symptom duration, and severity of symptoms; for disability, differences were tested according to sex and age.

The satisfactory improvement score was calculated as the difference in the current symptom score from the satisfactory improvement score. Scores are expressed as the mean ± standard deviation. If the difference between the initial response value and the satisfactory improvement score was negative, the response was judged to be unreliable and was excluded from the calculation. Differences in satisfactory improvement scores by respondent characteristics were tested using analysis of variance. For disability, the percentage of respondents who responded that they would be satisfied with an improvement of 80% or more was determined, and differences by sex and age were tested using the chi-square and Fisher’s exact tests.

The distribution of important factors of treatment according to respondent characteristics is expressed as the frequency and percentage (N, %), and differences by sex, age, symptom duration, and severity of symptoms were tested using the chi-square test. A multivariate logistic regression model was used to analyze factors that influenced what patients considered more important in treatment. Sex, age, onset, and severity were used as covariates. All estimates are expressed as adjusted odds ratio (OR) and 95% confidential interval (CI). The area under the curve (AUC) was presented to evaluate the goodness of fit for the logistic regression model.

Unmet medical needs and intention for treatment according to respondent characteristics are expressed as the frequency and percentage (N, %), and differences by sex and age were analyzed using the chi-square and Fisher’s exact tests.

For sensitivity analysis, the analysis only for patients diagnosed with LDH on MRI was performed. All statistical analyses were performed using R-4.2.1 for Windows (The R Foundation).

## 3. Results

### 3.1. Participants

**Figure 1:**
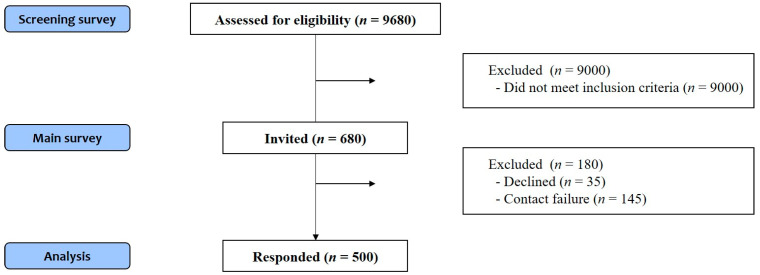
Flowchart of the study design.

In total, 9680 respondents were screened, with 9000 excluded for not meeting the inclusion criteria. Of the 680 respondents who met the inclusion criteria, 35 rejected the main survey and 145 failed to return it; thus, a total of 500 people responded to the main survey (Figure 1).

A total of 9680 patients were screened and 680 were invited to the main survey. Ultimately, 500 patients who completed the responses completely were analyzed.

### 3.2. Basic Respondent Characteristics

The basic characteristics of the respondents are presented in Table 1. Patients were equally distributed according to sex, with a male-to-female ratio of 1:1, and “clerks (office workers)” was the most common job type. The most common time of symptom onset was >12 months (45.4%), followed by ≥4 weeks to <12 weeks (23.2%), ≥12 weeks to <12 months (19.6%), and <4 weeks (11.8%). Regarding the presence of specific events, 199 respondents (46.4%) reported that they had experienced such an event. For triggers of symptom onset, the most common response was everyday activities (N = 53, 26.6%), followed by exercise (N = 40, 20.1%) and injury (N = 37, 18.6%). MRI was performed for 317 patients at the time of diagnosis. The distribution of characteristics of those 317 patients was similar to those of the entire group (Table 1).

### 3.3. LDH-Related Symptoms and Disability

**Figure 2:**
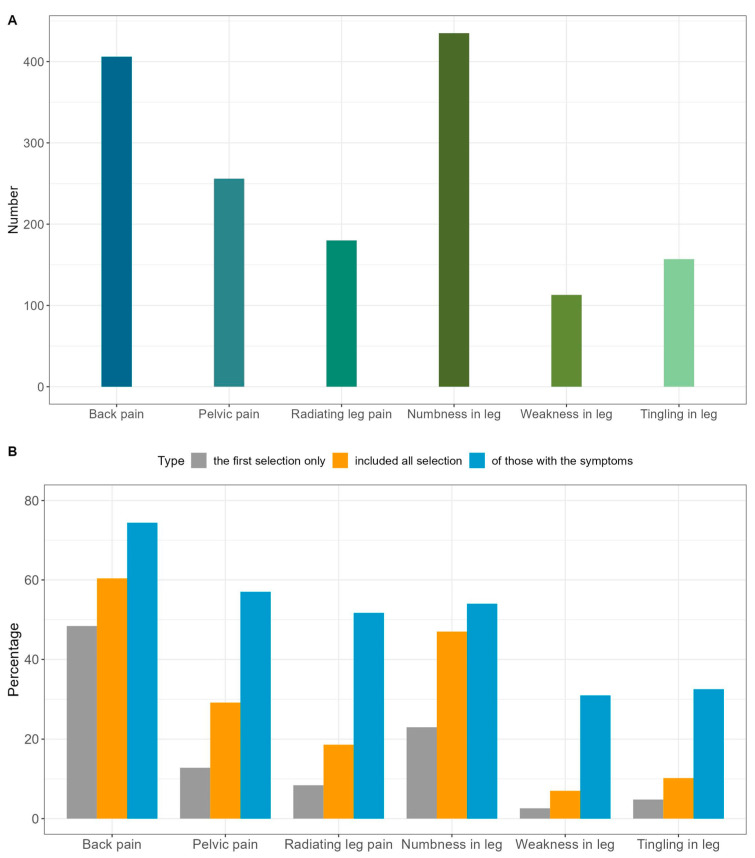
Lumbar intervertebral disc herniation symptoms and priority symptoms. (**A**) Number of respondents who reported experiencing each symptom. (**B**) Priority symptoms for improvement when only the first priority symptom is assessed, when the first and second priority symptoms are combined, and when the first and second priority symptoms are determined in those with the corresponding symptom (%).

LDH symptoms are shown in Figure 2A and Table 2. The most common symptoms were numbness in the leg (N = 435, 87.0%) and back pain (N = 406, 81.2%). The rates of back pain (84.6% vs. 77.9%, *p* = 0.069), pelvic pain (59.1% vs. 43.5%, *p* = 0.001), and radiating leg pain (40.1% vs. 32.0%, *p* = 0.074) were higher in women. Meanwhile, the rates of weakness in the leg (25.3% vs. 19.8%, *p* = 0.176) and tingling in the leg (35.6% vs. 27.1%, *p* = 0.053) were higher in men. When only patients diagnosed with MRI were analyzed, the rates of each symptom and the distribution according to characteristics were similar (Table S1).

**Figure 3:**
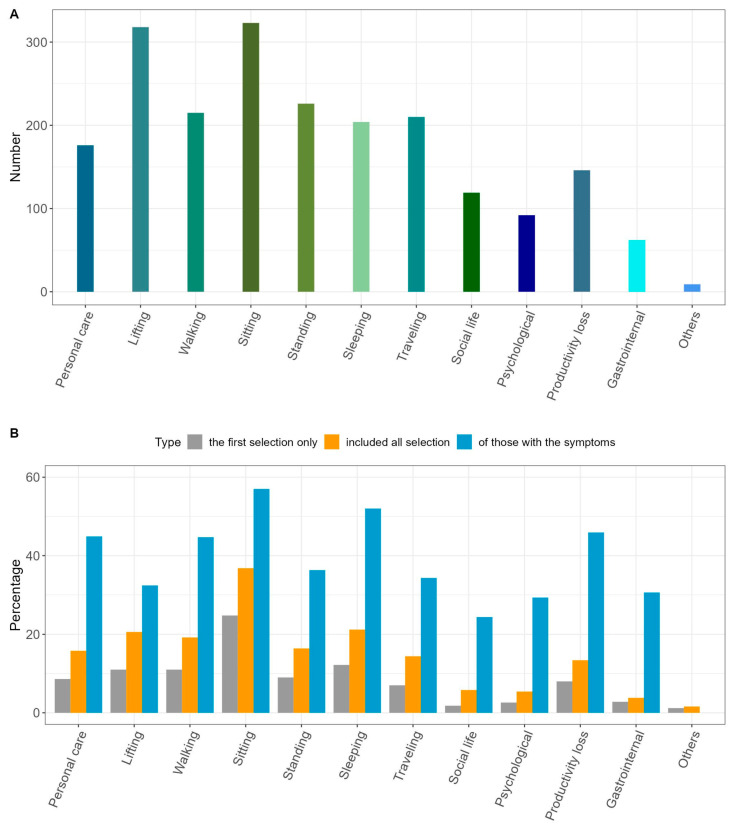
Disability due to lumbar disc herniation and type of priority disability for improvement. (**A**) Number of respondents with the disability. (**B**) Priority disability for improvement when only the first priority disability is assessed, when the first and second priority disabilities are combined, and when the first and second priority symptoms are determined in those with the corresponding symptom (%).

Types of LDH-related disability are displayed in Figure 3A and Table S2. The most common type of disability was difficulty in sitting (N = 323, 64.6%), followed by difficulty in lifting (N = 318, 63.6%). When analyzed by sex, the rates of difficulty in walking (49.4% vs. 36.8%, *p* = 0.006), sitting (70.9% vs. 58.5%, *p* = 0.005), standing (51.8% vs. 38.7%, *p* = 0.004), and sleeping (47.8% vs. 34.0%, *p* = 0.002) were higher in women. In addition, the rate of productivity loss (35.2% vs. 23.1%, *p* = 0.004) was significantly higher in men. When analyzed by age group, the rate of disability was higher in the older age group. Particularly, the older age group showed significantly higher rates of difficulty in lifting (*p* = 0.034), walking (*p* = 0.003), and traveling (*p* = 0.022); hampered social life (*p* = 0.013); and psychological problems (*p* < 0.001), productivity loss (*p* = 0.012), and gastrointestinal problems (*p* = 0.001). This tendency was similar for patients diagnosed via MRI (Table S3).

### 3.4. Severity of Symptoms and Disability of LDH

Symptom severity and disability are shown in Table S4 and Table S5, respectively. The average NRS score for each symptom was 5 points, and scores tended to be higher in the older age group, with no difference according to sex or time of onset. With respect to the severity of disability evaluated on the 5-point Likert scale, despite the differences, the respondents mostly reported mild disability (first and second level), and the rate of severe disability (fourth and fifth level) was low. The disabilities with more than a 10% severity rate included were lifting (15.4%), psychological problems (10.9%), productivity loss (14.4%), and gastrointestinal problems (12.9%). A high percentage of women reported severe difficulty in performing everyday activities, lifting, traveling, or social life; psychological problems; productivity loss; and heartburn. With respect to disability by age group, the proportion of those with severe symptoms and severe disabilities was higher in the older age group.

### 3.5. Priority Symptoms for Improvement

The priority symptoms for improvement are shown in Figure 2B and Table 3. The most prioritized symptom was back pain (N = 242, 48.4%), followed by numbness in the leg (N = 115, 23.0%) and pelvic pain (N = 64 12.8%). When the first and second priorities were combined, back pain (N = 302, 60.4%), numbness in the leg (N = 235, 47.0%), and pelvic pain (N = 146, 29.2%) were the most prioritized symptoms. In those with the corresponding symptoms, 74.4% of the patients with back pain, 57.0% of the patients with pelvic pain, and 54.0% of the patients with numbness in the leg responded that they hoped the corresponding symptom would improve.

Table 3 presents the priority symptoms for improvement by respondent characteristics. Priority for improvement in pelvic pain was higher in women than in men (35.2% vs. 23.3%, *p* = 0.005), while the priority for improvement in numbness in the leg was higher in men than in women (51.4% vs. 42.5%, *p* = 0.058).

For patients diagnosed via MRI, the most prioritized symptom was also back pain (N = 191, 61.2%), followed by numbness in the leg and pelvic pain. Sex differences were also similar (Table S6).

The respondent-defined average decrease in the NRS score for satisfactory symptom improvement is presented in Table 4. Although the score differed by symptom, the respondents answered overall that symptom improvement would be satisfactory if the average change in the NRS score was 3 or higher. Compared with men, women desired a significantly greater degree of improvement for back pain (2.9 ± 2.2 points vs. 3.4 ± 2.1 points) and numbness in the leg (2.4 ± 2.0 points vs. 3.1 ± 2.6 points). The patients whose symptom onset time was ≥12 weeks desired a greater degree of improvement than did those whose symptom onset time was <12 weeks, although the difference was not significant. The more severe the symptoms, the greater the level of improvement desired, and this trend was significant for all symptoms.

For patients diagnosed via MRI, the satisfactory average change in the NRS score was also 3 or higher. Additionally, female patients with longer onsets and patients with more severe symptoms tended to desire greater levels of improvement (Table S7).

### 3.6. Priority Disabilities for Improvement

The priority disabilities for improvement are shown in Figure 3B and Table S8. Among the disabilities identified as the first priority for improvement, discomfort in sitting had the highest priority (N = 124, 24.8%), followed by discomfort in sleeping (N = 61, 12.2%), lifting (N = 55, 11.0%), and walking (N = 55, 11.0%). When the first and second-priority disabilities were combined, discomfort in sitting remained the highest priority (N = 184, 36.8%), followed by discomfort in sleeping (N = 106, 21.2%), lifting (N = 103, 20.6%), and walking (N = 96, 19.2%). When priority was determined in those with the corresponding discomfort, the priority for improvement was the highest in discomfort in sitting (N = 184, 57.0%), followed by discomfort in sleeping (N = 106, 52.0%) and productivity loss (N = 67, 45.9%).

The type of disability requiring priority in improvement according to the respondent’s sex and age, with the first and second priority disabilities combined, is shown in Table S8. Compared with female respondents, more male respondents desired improvement in discomfort in traveling (19.8% vs. 8.9%, *p* = 0.001) and productivity loss (19.8% vs. 6.9%, *p* < 0.001). Meanwhile, more female respondents desired improvements in discomfort in walking (23.1% vs. 15.4%, *p* = 0.039) and sitting (42.9% vs. 30.8%, *p* = 0.007) compared with male respondents. With respect to differences by age, the responses differed for each item rather than showing a consistent trend. Most respondents in their 30s prioritized improvement in lifting, those in their 60s prioritized improvement in walking, those in their 20s prioritized improvement in sitting, and those in their 40s prioritized improvement in traveling. There was a similar tendency shown among those who only underwent MRI at diagnosis (Table S9).

The respondent-reported satisfactory degree of improvement for each disability type is presented in Table S10. Although there were differences according to the type of disability, approximately 75–90% of the respondents reported that an improvement of ≥80% would be satisfactory. For discomfort in sleeping, 91.5% of the respondents reported satisfactory improvement as ≥80% improvement. There were no differences in response by sex or age.

### 3.7. Important Factors in Treatment

**Figure 4:**
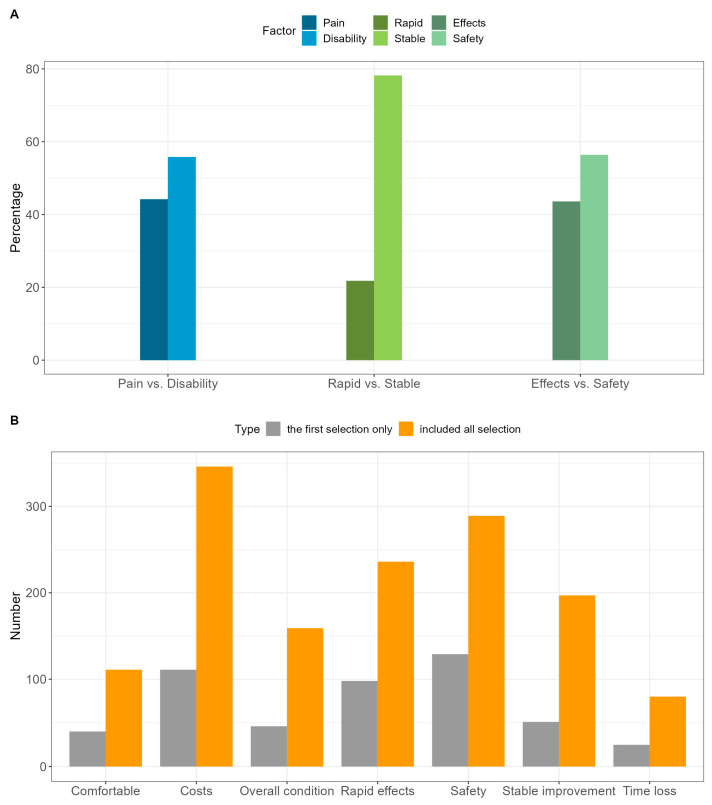
Important factors in treatment. (**A**) Treatment factor with higher priority between one of the two options. (**B**) Important factors in treatment.

The responses for important factors in treatment are shown in Figure 4 and Table 5. A higher proportion of respondents (N = 270, 55.8%) preferred improvement in functional disability over reduced pain. With respect to responses by sex, there were more male than female respondents who preferred improvement in functional disability (60.9% vs. 50.6%, *p* = 0.026). Regarding preference between rapid and stable effects without recurrence, the majority (N = 391, 78.2%) of respondents chose the latter; regarding preference by symptom onset time, more stable effects were desired over rapid effects. Between treatment effect and safety, the majority (N = 282, 56.4%) of the respondents preferred safety. Women placed a higher importance on safety than did men, and patients with chronic symptoms considered safety more important than did those with acute symptoms.

Patients diagnosed via MRI also preferred disability improvement, stable effects, and safety rather than pain reduction, rapid effects, and efficacy, respectively. Males preferred disability improvement more than female patients, and female patients and those with longer onsets tended to prefer safety (Table S11).

In factor analysis, the OR of female patients, using that of male patients as a reference, for the preference for functional improvement rather than pain reduction was 0.65 (95% CI 0.46 to 0.94, *p* = 0.021), indicating that females valued pain reduction over functional improvement compared to men (Table S12). The OR of patients with an onset of 12 months or longer, with patients with an onset of 4 weeks or less as a reference, for preference for a stable effect rather than a rapid effect was 2.57 (95% CI 1.33 to 4.96, *p* = 0.005), indicating that patients with a longer symptom onset desired a stable effect. Regarding the importance of safety over treatment effect, the OR of female to male was 1.45 (95% CI 1.01 to 2.09, *p* = 0.042), and the OR of patients with an onset of 12 months or more to patients with an onset of 4 weeks or less was 2.37 (95% CI 1.30 to 4.30, *p* = 0.005). Overall, female patients prioritized safer treatments compared to male patients, and those with long-term symptoms did so compared to patients with short-term symptoms. The AUCs of the multivariate logistic model for functional improvement, stable effects, and safety were 0.61, 0.62, and 0.61, respectively.

As the total number of samples decreased, the number of factors that had a significant effect decreased in the analysis conducted on patients who had undergone MRI at the time of diagnosis. However, the tendency for female patients to prefer pain reduction and safety compared to men and the tendency for patients with long duration of symptoms to prefer safety were consistent (Table S13).

For the important factors in treatment, when only the first priority was considered, the highest priorities were safety (N = 129, 25.8%) and cost (N = 111, 22.2%). When the top three factors were considered, cost (N = 346, 69.2%) and safety (N = 289, 57.8%) were the most important factors (Figure 4B).

### 3.8. Unmet Medical Needs and Intention to Treat

Responses regarding unmet medical needs and intention to treat are shown in Table S14. A considerable proportion of respondents (N = 232, 46.4%) reported not receiving sufficient medical care. The main reasons for insufficient medical care were time constraints (N = 158, 68.1%) and cost (N = 152, 65.5%). With respect to reasons for insufficient medical care by age, middle-aged respondents in their 40s selected time constraint as the main reason (N = 33, 82.5%), while younger respondents selected cost. The older age group reported a lack of effects as the main reason for not receiving more medical care.

When asked whether they would wish to receive continued care even after improvement in symptoms, a significant proportion of the patients (N = 306, 61.2%) expressed a desire for continued management. The preference for continued management was significantly higher in males (*p* = 0.001) and those who were in their 40s and older (*p* = 0.008). For the frequency and duration of medical care, the majority of the respondents selected 2–3 times (N = 232, 46.4%) or once per week (N = 157, 31.4%) and less than 1 h per treatment session (N = 391, 78.1%).

## 4. Discussion

This nationwide survey of patients with LDH elucidated outcomes that reflect real-world patient needs and concerns. The purpose of this study was to deepen the understanding of the current symptoms and needs of patients with LDH and consider the outcomes that can reflect these. As a cross-sectional design is used in descriptive studies to provide estimates of the prevalence of disease, traits such as people’s attitudes, knowledge or health behavior, or analytical studies to evaluate the associations between different parameters [28], we chose a cross-sectional questionnaire survey design for this investigation.

To the best of our knowledge, this study is the first to report a detailed analysis of the various types of symptoms and disabilities caused by LDH. While there have been previous studies on LDH symptoms and epidemiology, most of these investigated prevalence by sex and age [29] or objective outcome measures [30]. Few studies have been conducted regarding how the LDH experience differs by patient characteristics such as age and sex.

In this study, female patients selected pelvic pain as a priority symptom for improvement at a significantly higher rate than men, and the degree of satisfactory improvement was greater than that of men. In addition, among pain and disability, preference for pain relief was higher in women than in men. Although the reason for this sex difference is not clearly known, it has been established that female patients can be more sensitive to symptoms and more concerned about their health than men [31]. Another point to consider is that some female patients who participated in this survey were experiencing menopause. It is known that menopausal symptoms typically appear between the ages of 40 and 50 years. During this period, women experience various symptoms, including those that are psychological such as depression and anxiety, along with osteoporosis and genitourinary symptoms [32]. These psychological/physical effects of menopause may have influenced the survey results.

Additionally, few studies have investigated differences in symptoms by age, with the exception of those comparing pre- and postoperative outcomes [33,34]. One report showed that highly restricted positive straight leg raise test results and pain on coughing were commonly identified in the younger age group. Further, the prevalence of highly restricted straight leg raising test results decreased with increasing age, while the prevalence of severe reduction of walking capacity increased [35]. In contrast, the current study found no age-specific difference in walking. Further evidence is needed to establish the difference in LDH experience according to sex and age.

Back pain and numbness in the leg were top-priority symptoms for improvement. Particularly, 74.4% of the patients with back pain selected this pain as their first priority symptom for improvement. A typical symptom of LDH is sciatica [29], and many studies have used the NRS score of radiating leg pain as the primary outcome. However, the current study results indicate that in the real-world, many patients diagnosed with LDH suffer from back pain rather than leg pain. Yang Hao et al. [36] suggested that the high expression of inflammatory mediators in annulus fibrosus tears causes LBP in patients with LDH, and degenerative change with a high-intensity zone on MRI can be seen in patients with LDH and LBP. Other studies examined the performance of physical examination or evaluating the effects of discectomy in patients with LDH and LBP [37,38]. Therefore, it is thought that back pain should be treated as an outcome of equal importance to leg pain.

Concerning a satisfactory degree of improvement, female patients desired significantly higher degrees of improvement in back pain and numbness in the leg compared to male patients. In addition, patients with higher symptom severity desired a greater extent of improvement. Although there have been many studies with analysis of minimal clinically important difference (MCID) on the various outcomes of patients with LDH, MCID is typically expressed as a specific number and is not affected by the baseline value [39,40,41,42]. Therefore, judging patient improvement and the effectiveness of specific treatments based on MCID may introduce bias. As can be seen from the results of this study, patients with severe symptoms were only satisfied when they experienced considerable improvement, while those with mild symptoms were satisfied with small improvements. Although data were not shown, we found that when the degree of improvement was calculated as a percentage, the median level of desired improvement was approximately 50–60%. Therefore, to evaluate the status of improvement and the effectiveness of treatment, it would be preferable to use the percentage change from baseline or conduct a subgroup analysis by severity rather than the MCID score.

Among types of priority disabilities for improvement, many respondents selected improvements in sitting, lifting, sleeping, and walking as priorities. However, productivity loss was the second most common response in those who experienced it. Given that productivity loss is directly related to livelihood, it can be assumed that it is a critical problem for patients with related problems. In fact, normal economic activity is an important goal of treatment and is also an important socioeconomic factor. However, studies that evaluated the effectiveness of treatment generally did not include outcomes related to economic activity. In most cases, economic activity is only included as part of a cost calculation for economic evaluation. Thus, it is suggested that related outcomes, such as return to work and productivity loss, should be considered in clinical trials for patients with LDH.

Differences by sex were also identified in the types of priority disabilities for improvement. Male patients desired improvements in factors related to external activities such as traveling and productivity loss, while female patients desired more improvements in factors related to everyday activities such as walking and sitting. Instead of a consistent trend in differences by sex, there was no particular trend by age. Respondents in their 30s selected lifting disability as the first priority for improvement, those in their 60s selected walking, those in their 20s selected sitting, and those in their 40s selected traveling. 

More patients selected improvement in functional disability over that in pain reduction. However, the difference between the two was not large, and this tendency was greater in males. Pain and function are closely related factors, as they can act as both cause and effect and making dichotomous choices may have been difficult. This can be observed from the fact that the difference in preference for pain reduction and functional improvement was not large. Furthermore, pain and disability are different concepts, and the direction of changes in these outcomes is not always in agreement. In a study that analyzed the relationship among pain, disability, and quality of life in patients with LBP, improvement in pain did not lead to improvement in function or quality of life. Thus, it was concluded that outcome measures for pain and functional disability should be evaluated separately [43]. Therefore, there is no need to set only one as the primary outcome, and it would be prudent to set both outcomes as primary. Given that pain and function, as outcome measures, are independent domains, multiple comparison correction may not be necessary [44,45]. As no previous studies regarding men’s preferences for functional improvement have yet been reported, further related research is needed.

In addition, more patients selected a stable treatment effect over a rapid one and safety over treatment effects. Furthermore, patients with a longer duration of symptoms preferred treatment with stable effects over rapid effects and safety over treatment effects. Particularly, for the choice between rapid and stable treatment effects, approximately 80% of the patients selected stable effects, showing a clear pattern of preference. Nevertheless, outcome measures in clinical studies typically do not include the stability of treatment effects or recurrence. Most studies have focused on rapid effects such as survival analysis and degree of improvement within a set period. The findings of this study indicate that such outcomes may not reflect the real needs of the patients. Therefore, an outcome that could evaluate the stability of the treatment effect may be necessary, especially for clinical studies involving many patients with chronic conditions. Possible measures include the area under the curve within the follow-up period or the incidence of deterioration above a certain level after improvement. In this regard, strategies and methods for an effective evaluation of the stability of treatment effects should be cautiously considered.

Notably, although the difference was not large, many patients responded that safe treatment had high importance. In particular, women and patients with longer duration of symptoms showed a tendency to prefer safe treatment. Interestingly, patients do not necessarily prefer treatment with rapid effects just because their symptoms are severe. Although it may be perceived that patients with severe symptoms would prefer a treatment with rapid effects, the current study showed that in the real world, these patients also desired safe treatment more. However, in clinical trials of specific intervention for patients with LDH, safety is rarely treated as an important outcome. Therefore, particularly in the case of chronic patients, it is necessary to investigate adverse events more thoroughly.

In addition, approximately half of the respondents reported that they had unmet medical needs, and cost and time constraints were the major reasons for their unmet medical needs. In addition, more than 60% of patients responded that they preferred continued treatment despite symptom improvement. For the intention to receive treatment, most respondents preferred treatments once to three times weekly with a duration of less than 1 h per session. Ensuring treatment compliance is integral during clinical trials, and although the duration and frequency of treatment should be determined according to clinical judgment, there should be efforts to improve patient compliance by considering the patient’s intention for treatment as discussed above.

Overall, the results of this study should be interpreted with caution because of several limitations. First, as this survey was conducted in Korea, it is questionable whether the results can be applied to general patients with LDH. In particular, there is a possibility that the results related to factors considered important in treatment or unmet medical needs reflect distinct characteristics of Korea.

Secondly, as this study is an online survey, all data were self-reported. The same was true for the process of selecting respondents. That is, the diagnosis of LDH depended on the patient’s response. This may have affected the validity of the survey results. However, to increase the validity of the survey, responses with logical and formal errors between questions were deleted. Furthermore, this limitation can be observed in all survey studies, and a sample size of 500 is judged to guarantee the validity of the overall results despite the possibility of some unreliable responses. In this study, 2/3 of the patients responded that they had undergone an MRI at the time of diagnosis. The tendency of the overall results did not change, even in the sensitivity analysis on only patients who had had an MRI. These results further increase the validity of the actual LDH diagnosis of the respondents. The fact that 7% (680 out of 9680), the rate of satisfying the inclusion criteria in the screening questionnaire, was comparable to the Korean adult disc prevalence, may also be another rationale for validity.

Thirdly, although MRI is the most appropriate method to confirm LDH, we did not include whether or not respondents were diagnosed via MRI as part of the inclusion criteria. Instead, we attempted to specify the inclusion criteria as much as possible through including only patients with radiculopathy and those diagnosed with LDH by clinicians. This decision was made based on the North American Spine Society’s evidence-based guideline suggesting that manual muscle testing, sensory testing, and supine SLR tests are the gold standards for clinical diagnosis of LDH [5]. In contrast, as mentioned above, MRI was performed at the time of diagnosis in about 2/3 of the patients. Although specific results were not requested, it is thought that those with MRI readings, such as bulging, protrusion, extrusion, annular tear, and other conditions would have been diagnosed with LDH based on the general LDH diagnosis and classification criteria [46,47].

In addition, the possibility of a selection bias toward people who proficiently use mobile phones and apps could not be ruled out. Moreover, there may be concerns regarding whether 500 respondents can represent all patients with LDH and whether this is a sufficient number. Determining a sample size is difficult. Of course, larger sample sizes are better, but they entail remarkable effort and time. We determined the appropriate sample size for this study to be 500 based on the literature [20] along with our budget and time limitations. Furthermore, it is judged to have representativeness although statistical methods were not used for sample selection, as Opensurvey panels were distributed to various age groups across the country and the survey links were delivered after stratifying by sex and age.

Additionally, COVID-19, which has been prevalent since 2019, has had a significant impact on individuals and may have influenced respondents’ thoughts in some way. This point was not considered in this study, but it would be a better study if the impact of COVID-19 could also be explored in future research.

There were also limitations to the questions and questionnaire items themselves. A more in-depth analysis would have been possible if the survey had included more diverse items, such as body mass index, underlying disease, and genetic factors. In addition, although there may be points to consider in the clinical trial depending on the specific treatments, specific treatment that the patients received were not asked for in this study. However, this exploratory questionnaire was an analysis of outcomes that could be universally applied to any clinical trial for LDH patients. Nevertheless, there are cases where a specific outcome, such as reoperation, is the main outcome; depending on the type of intervention, the main outcomes are often related to pain and function regardless of the type of intervention in most clinical trials. Further, considering that an excess of complex questions may decrease the response rate and validity, and considering that this was an exploratory, descriptive study, we constructed the questionnaire with the minimal possible number of necessary items. In addition, MRI results were not asked for, as it was believed that most patients would not be aware of their MRI findings. If we had known the degree or type of disc herniation, we would have been able to conduct a more interesting analysis. However, the severity was classified depending on the patient’s clinical symptoms and subjective report. Furthermore, we attempted to include mental illness and intellectual disabilities as exclusion criteria, but these were ultimately not included because they were considered inappropriate in terms of application, and the validity of the response could not be guaranteed. Lastly, in survey studies, it is very important to present psychometric evidence of the questionnaire. However, due to the nature of this questionnaire, it was judged impossible to present commonly used evidence, so psychometric evidence was not presented. However, as a second option, we tried to secure the rationality of the survey in the process by explaining the process of creating the survey in as much detail as possible.

Despite these limitations, the present study provides an instrumental basis for designing clinical studies that reflect the real-world perspectives of patients with LDH by presenting detailed information on their symptoms, disability, and needs. Improvements in back pain, leg pain, sitting, and sleeping were prioritized, and safety, stable treatment effect, and functional recovery were desired. Clinical trials for LDH should be designed to reflect this real-world patient need.

As this study was an exploratory survey, the results should be interpreted with caution. We intend to plan a precisely designed registry study including a larger number of patients to overcome the limitations of this survey study and strengthen its validity and generalizability.

## Figures and Tables

**Table 1:** Basic respondent characteristics.

	All Respondents	MRI at Diagnosis
	N (%)	N (%)
Total	500 (100%)	317(100%)
Sex		
Male	253 (50.60%)	172 (54.3%)
Female	247 (49.40%)	145 (45.7%)
Age (years)		
Mean (mean ± SD)	44.37 ± 13.70	44.95 ± 13.32
Area of residence		
Seoul	167 (33.4%)	106 (33.44)
Capital area	173 (34.6%)	111 (35.0%)
Metropolitan Cities	86 (17.2%)	51 (16.1%)
Others	74 (14.8%)	49 (15.5%)
Occupation		
Managers	29 (5.8%)	19 (6.0%)
Professionals	66 (13.2%)	42 (13.3%)
Clerks	160 (32.0%)	101 (31.9%)
Service workers	56 (11.2%)	32 (10.1%)
Sales workers	15 (3.0%)	9 (2.8%)
Skilled agricultural, forestry, and fishery workers	5 (1.0%)	5 (1.6%)
Craft and related trades workers	11 (2.2%)	3 (0.9%)
Plant and machine operators and assemblers	6 (1.2%)	4 (1.3%)
Elementary occupations	13 (2.6%)	9 (2.8%)
Armed forces occupations	5 (1.0%)	4 (1.3%)
No occupation	134 (26.8%)	89 (28.1%)
Symptom onset time		
≤4 weeks	59 (11.8%)	31 (9.8%)
>4 to <12 weeks	116 (23.2%)	66 (20.8%)
≥12 week to <12 months	98 (19.6%)	58 (18.3%)
≥12 months	227 (45.4%)	162 (51.1%)
Presence of a trigger event (N = 429)		N = 273
Yes	199 (46.4%)	136 (49.8%)
No	230 (53.6%)	137 (50.2%)
Trigger event (N = 199)		N = 136
Lifting of a heavy object	32 (16.1%)	20 (14.7%)
Pregnancy, childbirth, childrearing	8 (4.0%)	5 (3.7%)
Physical exercise	40 (20.1%)	28 (20.6%)
Work	22 (11.1%)	13 (9.6%)
Injury (e.g., hurt/wounded, traffic accident)	37 (18.6%)	28 (20.6%)
Everyday activities	53 (26.6%)	35 (25.7%)
Others or unknown	7 (3.5%)	7 (5.1%)
Severity of symptoms		
Severe	147 (29.4%)	113 (35.6%)
Moderate to severe	178 (35.6%)	105 (33.1%)
Mild to moderate	175 (35.0%)	99 (31.2%)
MRI at diagnosis		
Yes	317 (63.4%)	-
No	137 (27.4%)	-
Can’t remember	46 (9.2%)	-

SD, standard deviation; MRI, magnetic resonance imaging.

**Table 2:** Symptom distribution by basic respondent characteristics.

	Back Pain	Pelvic Pain	Radiating Leg Pain	Numbness in the Leg	Weakness in the Leg	Tingling in the Leg
	N (%)	N (%)	N (%)	N (%)	N (%)	N (%)
Total (N = 500)	406 (81.2)	256 (51.2)	180 (36.0)	435 (87.0)	113 (22.6)	157 (31.4)
Sex						
Male (N = 253)	197 (77.9)	110 (43.5)	81 (32.0)	221 (87.4)	64 (25.3)	90 (35.6)
Female (N = 247)	209 (84.6)	146 (59.1)	99 (40.1)	214 (86.6)	49 (19.8)	67 (27.1)
*p*-value	0.069	0.001	0.074	0.917	0.176	0.053
Age group, years						
19–29 (n = 100)	85 (85.0)	47 (47.0)	30 (30.0)	86 (86.0)	29 (29.0)	27 (27.0)
30–39 (n = 100)	83 (83.0)	63 (63.0)	37 (37.0)	90 (90.0)	18 (18.0)	30 (30.0)
40–49 (n = 100)	80 (80.0)	42 (42.0)	33 (33.0)	88 (88.0)	16 (16.0)	28 (28.0)
50–59 (n = 100)	77 (77.0)	51 (51.0)	44 (44.0)	83 (83.0)	23 (23.0)	35 (35.0)
≥60 (n = 100)	81 (81.0)	53 (53.0)	36 (36.0)	88 (88.0)	27 (27.0)	37 (37.0)
*p*-value	0.661	0.044	0.311	0.649	0.128	0.465
Symptom onset time						
<4 weeks (n = 59)	45 (76.3)	33 (55.9)	22 (37.3)	51 (86.4)	14 (23.7)	16 (27.1)
≥4 to <12 weeks (n = 116)	96 (82.8)	68 (58.6)	44 (37.9)	103 (88.8)	35 (30.2)	42 (36.2)
≥12 weeks to <12 months (n = 98)	77 (78.6)	42 (42.9)	27 (27.6)	86 (87.8)	14 (14.3)	22 (22.5)
≥12 months (n = 227)	188 (82.8)	113 (49.8)	87 (38.3)	195 (85.9)	50 (22.0)	77 (33.9)
*p*-value	0.581	0.112	0.284	0.888	0.051	0.109
Severity						
Severe (n = 147)	126 (85.7)	91 (61.9)	70 (47.6)	128 (87.1)	52 (35.4)	68 (46.3)
Moderate to severe (n = 178)	150 (84.3)	95 (53.4)	62 (34.8)	159 (89.3)	30 (16.9)	43 (24.2)
Mild to moderate (n = 175)	130 (74.3)	70 (40.0)	48 (27.4)	148 (84.6)	31 (17.7)	46 (26.3)
*p*-value	0.014	<0.001	0.001	0.414	<0.001	<0.001

All percentage values were calculated with the N in each row as the denominator. Symptom severity is defined according to the numeric rating scale score as follows: ≥8, severe; 6–7, moderate to severe; ≤5, mild to moderate.

**Table 3:** Priority symptoms for improvement by respondent characteristics.

	Back Pain	Pelvic Pain	Radiating Leg Pain	Numbness in the Leg	Weakness in the Leg	Tingling in the Leg
	N (%)	N (%)	N (%)	N (%)	N (%)	N (%)
Total (N = 500)	302 (60.4)	146 (29.2)	93 (18.6)	235 (47.0)	35 (7.0)	51 (10.2)
Sex						
Male (n = 253)	149 (58.9)	59 (23.3)	47 (18.6)	130 (51.4)	22 (8.7)	31 (12.3)
Female (n = 247)	153 (61.9)	87 (35.2)	46 (18.6)	105 (42.5)	13 (5.3)	20 (8.1)
*p*-value	0.545	0.005	1.000	0.058	0.184	0.165
Age group, years						
19–29 (n = 100)	69 (69.0)	22 (22.0)	14 (14.0)	44 (44.0)	10 (10.0)	10 (10.0)
30–39 (n = 100)	60 (60.0)	36 (36.0)	20 (20.0)	49 (49.0)	5 (5.0)	11 (11.0)
40–49 (n = 100)	64 (64.0)	28 (28.0)	18 (18.0)	49 (49.0)	4 (4.0)	11 (11.0)
50–59 (n = 100)	55 (55.0)	25 (25.0)	24 (24.0)	49 (49.0)	7 (7.0)	12 (12.0)
≥60 (n = 100)	54 (54.0)	35 (35.0)	17 (17.0)	44 (44.0)	9 (9.0)	7 (7.0)
*p*-value	0.160	0.121	0.456	0.877	0.407	0.806
Symptom onset time						
<4 weeks (n = 59)	29 (49.2)	12 (20.3)	11 (18.6)	32 (54.2)	5 (8.5)	6 (10.2)
≥4 weeks to <12 weeks (n = 116)	73 (62.9)	38 (32.8)	24 (20.7)	52 (44.8)	12 (10.3)	12 (10.3)
≥12 weeks to <12 months (n = 98)	61 (62.2)	26 (26.5)	14 (14.3)	52 (53.1)	3 (3.1)	12 (12.2)
≥12 months (n = 227)	139 (61.2)	70 (30.8)	44 (19.4)	99 (43.6)	15 (6.6)	21 (9.3)
*p*-value	0.303	0.310	0.652	0.267	0.186	0.879
Severity						
Severe (n = 147)	89 (60.5)	51 (34.7)	34 (23.1)	66 (44.9)	11 (7.5)	19 (12.9)
Moderate to severe (n = 178)	112 (62.9)	57 (32.0)	34 (19.1)	81 (45.5)	9 (5.1)	18 (10.1)
Mild to moderate (n = 175)	101 (57.7)	38 (21.7)	25 (14.3)	88 (50.3)	15 (8.6)	14 (8.0)
*p*-value	0.606	0.023	0.124	0.555	0.417	0.347

All percentage values were calculated with the n in each row as the denominator. Symptom severity is defined according to the numeric rating scale score as follows: ≥8, severe; 6–7, moderate to severe; ≤5, mild to moderate.

**Table 4:** Respondent-defined decrease in the NRS score for satisfactory improvement.

	Back Pain	Pelvic Pain	Radiating Leg Pain	Numbness in the Leg	Weakness in the Leg	Tingling in the Leg
Total (N = 500)	3.1 ± 2.2	3.3 ± 2.3	3.0 ± 2.3	2.7 ± 2.3	2.3 ± 2.1	3.0 ± 2.5
Sex						
Male (n = 253)	2.9 ± 2.2	3.3 ± 2.3	2.7 ± 2.3	2.4 ± 2.0	2.4 ± 2.2	2.7 ± 2.6
Female (n = 247)	3.4 ± 2.1	3.3 ± 2.4	3.4 ± 2.2	3.1 ± 2.6	2.2 ± 2.1	3.4 ± 2.3
*p*-value	0.043	0.973	0.150	0.016	0.784	0.334
Age group, years						
19–29 (n = 100)	3.0 ± 2.0	3.8 ± 2.0	3.3 ± 2.7	2.8 ± 2.2	2.4 ± 1.8	2.7 ± 1.9
30–39 (n = 100)	3.2 ± 2.2	3.3 ± 2.8	3.3 ± 2.6	2.8 ± 2.0	1.2 ± 1.6	3.5 ± 3.2
40–49 (n = 100)	3.2 ± 2.2	2.9 ± 2.1	2.9 ± 2.1	2.8 ± 2.3	2.0 ± 2.2	2.8 ± 2.3
50–59 (n = 100)	2.9 ± 2.2	3.5 ± 2.3	3.1 ± 2.3	2.6 ± 2.6	2.2 ± 2.6	2.8 ± 2.3
≥60 (n = 100)	3.4 ± 2.3	3.0 ± 2.2	2.5 ± 1.7	2.6 ± 2.4	3.1 ± 2.4	3.1 ± 2.9
*p*-value	0.827	0.699	0.829	0.980	0.635	0.936
Symptom onset time						
<4 weeks (n = 59)	2.6 ± 2.2	3.1 ± 2.4	1.7 ± 2.5	2.8 ± 2.8	0.4 ± 0.5	3.8 ± 1.8
≥4 weeks to <12 weeks (n = 116)	2.8 ± 2.1	2.8 ± 1.8	3.3 ± 2.0	2.5 ± 1.7	1.8 ± 2.0	3.6 ± 2.7
≥12 weeks to <12 months (n = 98)	2.9 ± 2.1	3.9 ± 2.3	2.7 ± 1.5	2.6 ± 2.3	1.7 ± 0.6	2.4 ± 2.2
≥12 months (n = 227)	3.5 ± 2.2	3.3 ± 2.5	3.3 ± 2.5	2.8 ± 2.4	3.4 ± 2.2	2.8 ± 2.7
*p*-value	0.039	0.271	0.189	0.909	0.022	0.603
Severity						
Severe (n = 147)	4.1 ± 2.7	4.5 ± 2.6	3.8 ± 2.6	3.8 ± 2.7	2.7 ± 2.7	4.0 ± 3.0
Moderate to severe (n = 178)	3.2 ± 1.8	3.3 ± 1.9	3.2 ± 2.0	2.8 ± 2.1	3.4 ± 2.1	3.0 ± 2.1
Mild to moderate (n = 175)	2.1 ± 1.4	1.6 ± 1.3	1.7 ± 1.4	1.7 ± 1.5	1.3 ± 1.1	1.5 ± 0.8
*p*-value	<0.001	<0.001	0.001	<0.001	0.039	0.025

NRS; numeric rating scale.

**Table 5:** Important factors in treatment (dichotomous choice).

	Pain vs. Disability		Rapid vs. Stable Treatment Effect		Treatment Effect vs. Safety	
	Pain	Disability	Rapid Effects	Stable Effect	Treatment Effect	Safety
	N (%)	N (%)	N (%)	N (%)	N (%)	N (%)
Total (N = 500)	221 (44.2)	279 (55.8)	109 (21.8)	391 (78.2)	218 (43.6)	282 (56.4)
Sex						
Male (n = 253)	99 (39.1)	154 (60.9)	54 (21.3)	199 (78.7)	121 (47.8)	132 (52.2)
Female (n = 247)	122 (49.4)	125 (50.6)	55 (22.3)	192 (77.7)	97 (39.3)	150 (60.7)
*p*-value		0.026		0.887		0.066
Age group, years						
19–29 (n = 100)	52 (52.0)	48 (48.0)	24 (24.0)	76 (76.0)	45 (45.0)	55 (55.0)
30–39 (n = 100)	40 (40.0)	60 (60.0)	25 (25.0)	75 (75.0)	44 (44.0)	56 (16.0)
40–49 (n = 100)	40 (40.0)	60 (60.0)	26 (26.0)	74 (74.0)	37 (37.0)	63 (63.0)
50–59 (n = 100)	41 (41.0)	59 (59.0)	17 (17.0)	83 (83.0)	49 (49.0)	51 (51.0)
≥60 (n = 100)	48 (48.0)	52 (52.0)	17 (17.0)	83 (83.0)	43 (43.0)	57 (57.0)
*p*-value		0.298		0.328		0.548
Symptom onset time						
<4 weeks (n = 59)	26 (44.1)	33 (55.9)	20 (33.9)	39 (66.1)	35 (59.3)	24 (40.7)
≥4 weeks to <12 weeks (n = 116)	57 (49.1)	59 (50.9)	30 (25.9)	86 (74.1)	51 (44.0)	65 (56.0)
≥12 weeks to <12 months (n = 98)	32 (32.7)	66 (67.4)	22 (22.5)	76 (77.6)	44 (44.9)	54 (55.1)
≥12 months (n = 227)	106 (46.7)	121 (53.3)	37 (16.3)	190 (83.7)	88 (38.8)	139 (61.2)
*p*-value		0.071		0.017		0.043
Severity						
Severe (n = 147)	63 (42.9)	84 (57.1)	30 (20.4)	117 (79.6)	73 (49.7)	74 (50.3)
Moderate to severe (n = 178)	79 (44.4)	99 (55.6)	41 (23.0)	137 (77.0)	77 (43.3)	101 (56.7)
Mild to moderate (n = 175)	79 (45.1)	96 (54.9)	38 (21.7)	137 (78.3)	68 (38.9)	107 (61.1)
*p*-value		0.917		0.849		0.149

## Data Availability

The data presented in this study are available on request from the corresponding author. The data generated during the study are not publicly available due to privacy/ethical restrictions.

## Supplementary Materials

The following supporting information can be downloaded at: https://www.mdpi.com/article/10.3390/healthcare11182598/s1. File S1. Disability Severity Measurement Questionnaire; Table S1. Symptom distribution by basic respondent characteristics. (For patients diagnosed on MRI); Table S2. Types of disabilities due to lumbar disc herniation according to sex and age; Table S3. Types of disability due to lumbar disc herniation according to sex and age. (For patients diagnosed on MRI); Table S4. Numeric rating scale scores for each symptom by respondent characteristics; Table S5. Severity of disability according to sex and age; Table S6. Priority symptoms for improvement by respondent characteristics. (For patients diagnosed on MRI); Table S7. Respondent-defined decrease in NRS score for satisfactory improvement. (For patients diagnosed on MRI); Table S8. Priority disabilities for improvement; Table S9. Priority disabilities for improvement (for patients diagnosed on MRI); Table S10. Age and sex distribution of respondents reporting satisfactory improvement as more than 80% by type of disability; Table S11. Important factors in treatment (for patients diagnosed on MRI); Table S12. Factors that influence on important factors patients consider in treatment; Table S13. Factors that influence on important factors patients consider in treatment (for patients diagnosed on MRI); Table S14. Unmet medical needs and the desire for treatment.

## References

[1] Hoy D., Bain C., Williams G., March L., Brooks P., Blyth F., Woolf A., Vos T., Buchbinder R. (2012). A systematic review of the global prevalence of low back pain. Arthritis Rheum..

[2] World Health Organization (2014). Priority Diseases and Reasons for Inclusion.

[3] James S.L., Abate D., Abate K.H., Abay S.M., Abbafati C., Abbasi N., Abbastabar H., Abd-Allah F., Abdela J., Abdelalim A. (2018). Global, regional, and national incidence, prevalence, and years lived with disability for 354 diseases and injuries for 195 countries and territories, 1990–2017: A systematic analysis for the Global Burden of Disease Study 2017. Lancet.

[4] Freburger J.K., Holmes G.M., Agans R.P., Jackman A.M., Darter J.D., Wallace A.S., Castel L.D., Kalsbeek W.D., Carey T.S. (2009). The rising prevalence of chronic low back pain. Arch. Intern. Med..

[5] Amin R.M., Andrade N.S., Neuman B.J. (2017). Lumbar Disc Herniation. Curr. Rev. Musculoskelet. Med..

[6] Schoenfeld A.J., Weiner B.K. (2010). Treatment of lumbar disc herniation: Evidence-based practice. Int. J. Gen. Med..

[7] Will J.S., Bury D.C., Miller J.A. (2018). Mechanical low back pain. Am. Fam. Physician.

[8] Axén I., Leboeuf-Yde C. (2013). Trajectories of low back pain. Best Pract. Res. Clin. Rheumatol..

[9] Hawker G.A., Mian S., Kendzerska T., French M. (2011). Measures of adult pain: Visual analog scale for pain (vas pain), numeric rating scale for pain (nrs pain), mcgill pain questionnaire (mpq), short-form mcgill pain questionnaire (sf-mpq), chronic pain grade scale (cpgs), short form-36 bodily pain scale (sf-36 bps), and measure of intermittent and constant osteoarthritis pain (icoap). Arthritis Care Res..

[10] Jeon C.-H., Kim D.-J., Kim D.-J., Lee H.-M., Park H.-J. (2005). Cross-cultural adaptation of the Korean version of the Oswestry Disability Index (ODI). J. Korean Soc. Spine Surg..

[11] Kim D.-Y., Lee S.-H., Lee H.-Y., Lee H.-J., Chang S.-B., Chung S.-K., Kim H.-J. (2005). Validation of the Korean version of the oswestry disability index. Spine.

[12] Stratford P.W., Binkley J.M., Riddle D.L., Guyatt G.H. (1998). Sensitivity to change of the Roland-Morris back pain questionnaire: Part 1. Phys. Ther..

[13] Baker A.D. (2013). A study of the natural history of back pain: Part I: Development of a reliable and sensitive measure of disability in low-back pain. Classic Papers in Orthopaedics.

[14] Monticone M., Baiardi P., Vanti C., Ferrari S., Pillastrini P., Mugnai R., Foti C. (2012). Responsiveness of the Oswestry Disability Index and the Roland Morris Disability Questionnaire in Italian subjects with sub-acute and chronic low back pain. Eur. Spine J..

[15] Iorio-Morin C., Dea N. (2018). Surgical versus Nonoperative Treatment for Lumbar Disc Herniation: The Spine Patient Outcomes Research Trial (SPORT): A Randomized Trial. 50 Landmark Papers.

[16] Peul W.C., Van Houwelingen H.C., van den Hout W.B., Brand R., Eekhof J.A., Tans J.T., Thomeer R.T., Koes B.W. (2007). Surgery versus prolonged conservative treatment for sciatica. N. Engl. J. Med..

[17] Xu J., Ding X., Wu J., Zhou X., Jin K., Yan M., Ma J., Wu X., Ye J., Mo W. (2020). A randomized controlled study for the treatment of middle-aged and old-aged lumbar disc herniation by Shis spine balance manipulation combined with bone and muscle guidance. Medicine.

[18] Kreiner D.S., Hwang S.W., Easa J.E., Resnick D.K., Baisden J.L., Bess S., Cho C.H., DePalma M.J., Dougherty II P., Fernand R. (2014). An evidence-based clinical guideline for the diagnosis and treatment of lumbar disc herniation with radiculopathy. Spine J..

[19] Andersson G., Brown M.D., Dvorak J., Herzog R.J., Kambin P., Malter A., McCulloch J.A., Saal J.A., Spratt K.F., Weinstein J.N. (1996). Consensus summary of the diagnosis and treatment of lumbar disc herniation. Spine.

[20] Gunawan J., Marzilli C., Aungsuroch Y. (2021). Establishing appropriate sample size for developing and validating a questionnaire in nursing research. Belitung Nurs. J..

[21] Cummins J., Lurie J.D., Tosteson T., Hanscom B., Abdu W.A., Birkmeyer N.J., Herkowitz H., Weinstein J. (2006). Descriptive epidemiology and prior healthcare utilization of patients in the spine patient outcomes research trial’s (sport) three observational cohorts: Disc herniation, spinal stenosis and degenerative spondylolisthesis. Spine.

[22] Ma D., Liang Y., Wang D., Liu Z., Zhang W., Ma T., Zhang L., Lu X., Cai Z. (2013). Trend of the incidence of lumbar disc herniation: Decreasing with aging in the elderly. Clin. Interv. Aging.

[23] Kim M.-H., Lee Y.J., Shin J.-S., Lee J., Jeong H., Kim M.-R., Park S.-M., Go U., Kim S.-M., Kim J.-Y. (2017). The long-term course of outcomes for lumbar intervertebral disc herniation following integrated complementary and alternative medicine inpatient treatment: A prospective observational study. Evid.-Based Complement. Altern. Med..

[24] Kim D., Choi Y., Yang C., Lee Y.J., Han C.-H., Ha I. (2022). Influencing Factors for Choosing Korean Medicine Treatments after Spinal Surgery or Spinal Surgery Recommendation in Patients with Spinal Pain: A Semi-Structured Interview Study. Complement. Med. Res..

[25] Solodiuk J.C., Scott-Sutherland J., Meyers M., Myette B., Shusterman C., Karian V.E., Harris S.K., Curley M.A. (2010). Validation of the Individualized Numeric Rating Scale (INRS): A pain assessment tool for nonverbal children with intellectual disability. Pain.

[26] Sham P.C., Curtis D. (1995). Monte Carlo tests for associations between disease and alleles at highly polymorphic loci. Ann Hum Genet.

[27] Hope A.C. (1968). A simplified Monte Carlo significance test procedure. J. R. Stat. Soc. Ser. B Stat. Methodol..

[28] Kesmodel U.S. (2018). Cross-sectional studies–What are they good for?. Acta Obstet. Gynecol. Scand..

[29] Vialle L.R., Vialle E.N., Henao J.E.S., Giraldo G. (2010). Lumbar disc herniation. Rev. Bras. Ortop..

[30] Kortelainen P., Puranen J., Koivisto E., Lähde S. (1985). Symptoms and signs of sciatica and their relation to the localization of the lumbar disc herniation. Spine.

[31] Green C.A., Pope C.R. (1999). Gender, psychosocial factors and the use of medical services: A longitudinal analysis. Soc. Sci. Med..

[32] Greendale G.A., Lee N.P., Arriola E.R. (1999). The menopause. Lancet.

[33] Siccoli A., Schröder M.L., Staartjes V.E. (2021). Association of age with incidence and timing of recurrence after microdiscectomy for lumbar disc herniation. Eur. Spine J..

[34] Strömqvist F., Strömqvist B., Jönsson B., Karlsson M.K. (2017). Surgical treatment of lumbar disc herniation in different ages—Evaluation of 11,237 patients. Spine J..

[35] Jönsson B., Strömqvist B. (1995). Influence of age on symptoms and signs in lumbar disc herniation. Eur. Spine J..

[36] Yang H., Liu H., Li Z., Zhang K., Wang J., Wang H., Zheng Z. (2015). Low back pain associated with lumbar disc herniation: Role of moderately degenerative disc and annulus fibrous tears. Int. J. Clin. Exp. Med..

[37] Leinonen V., Kankaanpää M., Luukkonen M., Kansanen M., Hänninen O., Airaksinen O., Taimela S. (2003). Lumbar paraspinal muscle function, perception of lumbar position, and postural control in disc herniation-related back pain. Spine.

[38] Van Der Windt D.A., Simons E., Riphagen I.I., Ammendolia C., Verhagen A.P., Laslett M., Devillé W., Deyo R.A., Bouter L.M., de Vet H.C. (2010). Physical examination for lumbar radiculopathy due to disc herniation in patients with low-back pain. Cochrane Database Syst. Rev..

[39] Heo D.H., Lee D.C., Kim H.S., Park C.K., Chung H. (2021). Clinical results and complications of endoscopic lumbar interbody fusion for lumbar degenerative disease: A meta-analysis. World Neurosurg..

[40] Azimi P., Yazdanian T., Benzel E.C. (2018). Determination of minimally clinically important differences for JOABPEQ measure after discectomy in patients with lumbar disc herniation. J. Spine Surg..

[41] Copay A.G., Glassman S.D., Subach B.R., Berven S., Schuler T.C., Carreon L.Y. (2008). Minimum clinically important difference in lumbar spine surgery patients: A choice of methods using the Oswestry Disability Index, Medical Outcomes Study questionnaire Short Form 36, and pain scales. Spine J..

[42] Sharma S.B., Lin G.-X., Jabri H., Sidappa N.D., Song M.S., Choi K.C., Kim J.-S. (2019). Radiographic and clinical outcomes of huge lumbar disc herniations treated by transforaminal endoscopic discectomy. Clin. Neurol. Neurosurg..

[43] Kovacs F.M., Abraira V., Zamora J., Teresa Gil del Real M., Llobera J., Fernández C., Bauza J.R., Bauza K., Coll J., Cuadri M. (2004). Correlation between pain, disability, and quality of life in patients with common low back pain. Spine.

[44] Peul W.C., van den Hout W.B., Brand R., Thomeer R.T., Koes B.W., Group L.-T.H.S.I.P.S. (2008). Prolonged conservative care versus early surgery in patients with sciatica caused by lumbar disc herniation: Two year results of a randomised controlled trial. BMJ.

[45] Skargren E.I., Carlsson P.G., Öberg B.E. (1998). One-year follow-up comparison of the cost and effectiveness of chiropractic and physiotherapy as primary management for back pain: Subgroup analysis, recurrence, and additional health care utilization. Spine.

[46] Milette P.C. (1997). The proper terminology for reporting lumbar intervertebral disk disorders. AJNR Am. J. Neuroradiol..

[47] Milette P.C. (2000). Classification, diagnostic imaging, and imaging characterization of a lumbar herniated disk. Radiol. Clin..

