# Supplementary material for: Are Primary Outcomes Really Primary? An Exploratory Cross-Sectional Nationwide Web-Based Survey Study for Outcomes Reflecting Real Symptoms and Needs of Patients with Lumbar Disc Herniation
Source: Healthcare (Basel). 2023 Sep 21;11(18):2598. doi: 10.3390/healthcare11182598 (PMC10531434; doi:10.3390/healthcare11182598)
Supplement: Supplementary file 1 [file healthcare-11-02598-s001.zip › S10_Table.pdf]

Table S10. Age and sex distribution of respondents reporting satisfactory improvement as more than 80% by type of disability

	Total	Sex			Age (years)					
		Male	Female	*P*-value	19-29	30-39	40-49	50-59	≥60	*P*-value
Personal care (N=79)	65 (82.3)	29 (78.4)	36 (85.7)	0.578	11 (91.7)	13 (81.2)	12 (85.7)	16 (72.7)	13 (86.7)	0.748
Lifting (N=103)	85 (82.5)	47 (82.46)	38 (82.61)	1.000	14 (93.3)	30 (88.2)	17 (73.9)	9 (64.3)	15 (88.2)	0.177
Walking (N=96)	83 (86.5)	33 (84.6)	50 (87.7)	0.894	11 (100.0)	14 (87.5)	21 (95.5)	13 (76.5)	24 (80.0)	0.231
Sitting (N=184)	158 (85.9)	64 (82.1)	94 (88.7)	0.289	47 (88.7)	33 (91.7)	29 (78.4)	22 (78.6)	27 (90.0)	0.338
Standing (N=82)	73 (89.0)	32 (94.1)	41 (85.4)	0.294	22 (91.7)	11 (91.7)	11 (91.7)	13 (76.5)	16 (94.1)	0.584
Sleep (N=106)	97 (91.5)	41 (87.2)	56 (94.9)	0.181	25 (89.3)	20 (95.2)	20 (95.2)	14 (77.8)	18 (100.0)	0.188
Travelling (N=72)	65 (90.3)	45 (90.0)	20 (90.9)	1.000	7 (87.5)	10 (90.9)	19 (90.5)	15 (88.2)	14 (93.3)	1.000
Social life (N=29)	23 (79.3)	14 (77.8)	9 (81.8)	1.000	3 (75.0)	4 (100.0)	6 (75.0)	4 (57.1)	6 (100.0)	0.331
Psychological (N=27)	20 (74.1)	13 (81.2)	7 (63.6)	0.391	1 (33.3)	6 (100.0)	5 (100.0)	4 (57.1)	4 (66.7)	0.093
Productivity loss (N=67)	58 (86.6)	42 (84.0)	16 (94.1)	0.430	7 (87.5)	16 (84.2)	10 (100.0)	16 (84.2)	9 (81.8)	0.818
Gastrointestinal (N=19)	13 (68.4)	8 (80.0)	5 (55.6)	0.350	1 (33.3)	0 (0.0)	0 (0.0)	5 (83.3)	7 (87.5)	0.056
Others (N=8)	6 (75.0)	3 (100.0)	3 (60.0)	0.464	4 (100.0)	-	2 (100.0)	0 (0.0)	0 (0.0)	0.071
