# Supplementary material for: Are Primary Outcomes Really Primary? An Exploratory Cross-Sectional Nationwide Web-Based Survey Study for Outcomes Reflecting Real Symptoms and Needs of Patients with Lumbar Disc Herniation
Source: Healthcare (Basel). 2023 Sep 21;11(18):2598. doi: 10.3390/healthcare11182598 (PMC10531434; doi:10.3390/healthcare11182598)
Supplement: Supplementary file 1 [file healthcare-11-02598-s001.zip › S11_Table.pdf]

Table S11. Important factors in treatment. (For patients diagnosed on MRI)

	**Pain vs disability**		**Rapid vs stable treatment effect**		**Treatment effect vs safety**	
	pain	disability	Rapid effects	Stable effect	Treatment effect	safety
	N (%)	N (%)	N (%)	N (%)	N (%)	N (%)
**Total (N=317)**	146 (46.1%)	171 (53.9%)	59 (18.6%)	258 (81.4%)	137 (43.2%)	180 (56.8%)
**Sex**						
Male (n=172)	70 (40.7%)	102 (59.3%)	29 (16.9%)	143 (83.1%)	81 (47.1%)	91 (52.9%)
Female (n=145)	76 (52.4%)	69 (47.6%)	30 (20.7%)	115 (79.3%)	56 (38.6%)	89 (61.4%)
P-value		0.049		0.467		0.161
**Age group, years**						
19-29 (n=57)	29 (50.9%)	28 (49.1%)	10 (17.5%)	47 (82.5%)	24 (42.1%)	33 (57.9%)
30-39 (n=59)	24 (40.7%)	35 (59.3%)	10 (16.9%)	49 (83.1%)	24 (40.7%)	35 (59.3%)
40-49 (n=68)	31 (45.6%)	37 (54.4%)	16 (23.5%)	52 (76.5%)	26 (38.2%)	42 (61.8%)
50-59 (n=70)	28 (40.0%)	42 (60.0%)	12 (17.1%)	58 (82.9%)	36 (51.4%)	34 (48.6%)
≥60 (n=63)	34 (54.0%)	29 (46.0%)	11 (17.5%)	52 (82.5%)	27 (42.9%)	36 (57.1%)
P-value		0.427		0.846		0.592
**Symptom onset time**						
<4 weeks (n=31)	16 (51.6%)	15 (48.4%)	5 (16.1%)	26 (83.9%)	20 (64.5%)	11 (35.5%)
≥4 weeks to <12 weeks (n=66)	33 (50.0%)	33 (50.0%)	18 (27.3%)	48 (72.7%)	28 (42.4%)	38 (57.6%)
≥12 weeks to <12 months (n=58)	18 (31.0%)	40 (69.0%)	14 (24.1%)	44 (75.9%)	25 (43.1%)	33 (56.9%)
≥12 months (n=162)	79 (48.8%)	83 (51.2%)	22 (13.6%)	140 (86.4%)	64 (39.5%)	98 (60.5%)
P-value		0.009		0.064		0.084
**Severity**						
Severe (n=113)	48 (42.5%)	65 (57.5%)	20 (17.7%)	93 (82.3%)	55 (48.7%)	58 (51.3%)
Moderate to severe (n=105)	53 (50.5%)	52 (49.5%)	21 (20.0%)	84 (80.0%)	39 (37.1%)	66 (62.9%)
Mild to moderate (n=99)	45 (45.5%)	54 (54.5%)	18 (18.2%)	81 (81.8%)	43 (43.4%)	56 (56.6%)
P-value		0.491		0.901		0.229
