# Supplementary material for: Are Primary Outcomes Really Primary? An Exploratory Cross-Sectional Nationwide Web-Based Survey Study for Outcomes Reflecting Real Symptoms and Needs of Patients with Lumbar Disc Herniation
Source: Healthcare (Basel). 2023 Sep 21;11(18):2598. doi: 10.3390/healthcare11182598 (PMC10531434; doi:10.3390/healthcare11182598)
Supplement: Supplementary file 1 [file healthcare-11-02598-s001.zip › S12_Table.pdf]

Table S12. Factors that influence on important factors patients consider in treatment

	Disability rather pain		Stable effects rather rapid effects		Safety rather treatment effects	
	OR	P value	OR	P value	OR	P value
**Sex (ref=Male)**						
Female (n=247)	0.65 (0.46-0.94)	0.021	0.95 (0.62-1.47)	0.820	1.45 (1.01-2.09)	0.042
**Age group, years (ref=19-29)**						
30-39 (n=100)	1.68 (0.95-2.97)	0.089	0.93 (0.48-1.80)	0.835	1.03 (0.58-1.81)	0.932
40-49 (n=100)	1.52 (0.85-2.72)	0.089	0.80 (0.41-1.56)	0.510	1.31 (0.73-2.37)	0.363
50-59 (n=100)	1.53 (0.86-2.71)	0.120	1.44 (0.71-2.93)	0.314	0.86 (0.48-1.53)	0.606
≥60 (n=100)	1.24 (0.70-2.21)	0.572	1.33 (0.65-2.73)	0.430	1.03 (0.58-1.84)	0.920
**Symptom onset time (ref= <4 weeks)**						
≥4 weeks to <12 weeks (n=116)	0.73 (0.38-1.39)	0.526	1.48 (0.74-2.95)	0.270	1.99 (1.04-3.80)	0.038
≥12 weeks to <12 months (n=98)	1.47 (0.74-2.93)	0.153	1.89 (0.90-3.97)	0.092	1.77 (0.90-3.48)	0.097
≥12 months (n=227)	0.86 (0.48-1.55)	0.718	2.57 (1.33-4.96)	0.005	2.37 (1.30-4.30)	0.005
**Severity (ref=mild to moderate)**						
Severe (n=147)	1.11 (0.72-1.72)	0.886	0.94 (0.56-1.58)	0.829	0.86 (0.55-1.33)	0.491
Moderate to severe (n=178)	1.15 (0.73-1.82)	0.681	0.99 (0.57-1.72)	0.970	0.64 (0.40-1.01)	0.054
**AUC**	0.61		0.62		0.61	
