# Supplementary material for: Are Primary Outcomes Really Primary? An Exploratory Cross-Sectional Nationwide Web-Based Survey Study for Outcomes Reflecting Real Symptoms and Needs of Patients with Lumbar Disc Herniation
Source: Healthcare (Basel). 2023 Sep 21;11(18):2598. doi: 10.3390/healthcare11182598 (PMC10531434; doi:10.3390/healthcare11182598)
Supplement: Supplementary file 1 [file healthcare-11-02598-s001.zip › S13_Table.pdf]

Table S13. Factors that influence on important factors patients consider in treatment. (For patients diagnosed on MRI)

	Disability rather pain		Stable effects rather rapid effects		Safety rather treatment effects	
	OR	P value	OR	P value	OR	P value
**Sex (ref=Male)**						
Female (n=145)	0.64 (0.40-1.01)	0.054	0.74 (0.41-1.32)	0.302	1.41 (0.89-2.24)	0.143
**Age group, years (ref=19-29)**						
30-39 (n=59)	1.47 (0.69-3.12)	0.32	1.15 (0.43-3.09)	0.785	1.02 (0.47-2.19)	0.966
40-49 (n=68)	1.02 (0.49-2.15)	0.95	0.74 (0.29-1.87)	0.521	1.13 (0.53-2.41)	0.75
50-59 (n=70)	1.41 (0.68-2.92)	0.359	1.08 (0.42-2.79)	0.876	0.66 (0.32-1.38)	0.271
≥60 (n=63)	0.89 (0.42-1.87)	0.751	0.89 (0.33-2.37)	0.812	0.94 (0.44- 2.02)	0.876
**Symptom onset time (ref= <4 weeks)**						
≥4 weeks to <12 weeks (n=116)	0.95 (0.39-2.29)	0.906	0.50 (0.16- 1.55)	0.233	2.75 (1.11-6.85)	0.03
≥12 weeks to <12 months (n=98)	2.18 (0.86-5.54)	0.1	0.63 (0.19- 2.01)	0.431	2.64 (1.03-6.75)	0.043
≥12 months (n=227)	1.10 (0.50-2.41)	0.82	1.26 (0.43-3.71)	0.672	3.05 (1.34-6.97)	0.008
**Severity (ref=mild to moderate)**						
Severe (n=147)	0.85 (0.48-1.50)	0.569	0.87 (0.42-1.81)	0.718	1.43 (0.79-2.56)	0.234
Moderate to severe (n=178)	1.23 (0.70-2.18)	0.475	1.05 (0.51-2.19)	0.888	0.81 (0.46-1.43)	0.468
**AUC**	0.62		0.64		0.62	
