# Supplementary material for: Are Primary Outcomes Really Primary? An Exploratory Cross-Sectional Nationwide Web-Based Survey Study for Outcomes Reflecting Real Symptoms and Needs of Patients with Lumbar Disc Herniation
Source: Healthcare (Basel). 2023 Sep 21;11(18):2598. doi: 10.3390/healthcare11182598 (PMC10531434; doi:10.3390/healthcare11182598)
Supplement: Supplementary file 1 [file healthcare-11-02598-s001.zip › S1_File.pdf]

Supplementary File S1. Disability Severity Measurement Questionnaire

1. **Personal care**

1) I can look after myself normally but it causes extra pain
2) It is painful to look after myself and I am slow and careful
3) I need some help but manage most of my personal care
4) I need help every day in most aspects of self care
5) I don't get dressed, I experience difficulty and stay in bed

2. **Lifting**

1) I can lift heavy weights but it causes extra pain
2) Pain prevents me from lifting heavy weights off the floor, but I can manage if they are conveniently positioned, i.e., on a table
3) Pain prevents me from lifting heavy weights, but I can manage light to medium weights if they are conveniently positioned
4) I can lift very light weights
5) I cannot lift or carry anything at all

3. **Walking**

1) Pain prevents me from walking more than one mile
2) Pain prevents me from walking more than ½ mile
3) Pain prevents me from walking more than ¼ mile
4) I can only walk using a stick or crutches
5) I am in bed most of the time and have to crawl to the toilet

4. **Sitting**

1) I can only sit in my favorite chair as long as I like
2) Pain prevents me from sitting more than one hour
3) Pain prevents me from sitting more than ½ hour
4) Pain prevents me from sitting more than 10 minutes
5) Pain prevents me from sitting at all

5. **Standing**

1) I can stand as long as I want but it causes me extra pain
2) Pain prevents me from standing for more than one hour
3) Pain prevents me from standing for more than 30 minutes
4) Pain prevents me from standing for more than 10 minutes
5) Pain prevents me from standing at all

6. **Sleeping**

1) I can sleep well only by using medication
2) Even when I take medication, I have less than 6 hrs sleep
3) Even when I take medication, I have less than 4 hrs sleep
4) Even when I take medication, I have less than 2 hrs sleep
5) Pain prevents me from sleeping at all

7. **Traveling:**

1) I can travel anywhere but it causes me extra pain
2) Pain is bad, but I manage journeys over 2 hours
3) Pain restricts me to journeys of less than 1 hour
4) Pain restricts me to short necessary journeys under 30 minutes
5) Pain prevents me from traveling except to the doctor or hospital

8. Social life

1) My social life is normal but increases the degree of pain
2) Pain has no significant effect on my social life apart from limiting my more energetic interests, i.e., dancing, etc.
3) Pain has restricted my social life and I do not go out as often
4) Pain has restricted my social life to my home
5) I have no social life because of pain

9. Psychological problem

1) I am a little anxious or depressed
2) I am slightly anxious or depressed
3) I am moderately anxious or depressed
4) I am severely anxious or depressed
5) I am extremely anxious or depressed

10. Productivity loss

1) Productivity loss is less than 20%
2) Productivity loss is 20% to 50%
3) Productivity loss is over 50%
4) In the past month, there was a gap in economic activities (annual leave, absenteeism) due to pain and discomfort
5) I have not been able to return to work

11. Gastrointestinal problems

1) Little discomfort
2) Slight discomfort
3) Moderate discomfort
4) Severe discomfort
5) Extreme discomfort
