# Supplementary material for: Are Primary Outcomes Really Primary? An Exploratory Cross-Sectional Nationwide Web-Based Survey Study for Outcomes Reflecting Real Symptoms and Needs of Patients with Lumbar Disc Herniation
Source: Healthcare (Basel). 2023 Sep 21;11(18):2598. doi: 10.3390/healthcare11182598 (PMC10531434; doi:10.3390/healthcare11182598)
Supplement: Supplementary file 1 [file healthcare-11-02598-s001.zip › S1_Table.pdf]

Table S1. Symptom distribution by basic respondent characteristics. (For patients diagnosed on MRI)

	**Back pain**	**Pelvic pain**	**Radiating leg pain**	**Numbness in the leg**	**Weakness in the leg**	**Tingling in the leg**
	N(%)	N(%)	N(%)	N(%)	N(%)	N(%)
**Total (n=317)**	262 (82.6%)	171 (53.9%)	128 (40.4%)	275 (86.8%)	79 (24.9%)	107 (33.8%)
**Sex**						
Male (n=172)	139 (80.8%)	85 (49.4%)	65 (37.8%)	149 (86.6%)	49 (28.5%)	67 (39.0%)
Female (n=145)	123 (84.8%)	86 (59.3%)	63 (43.4%)	126 (86.9%)	30 (20.7%)	40 (27.6%)
P-value	0.429	0.100	0.364	1.000	0.142	0.044
**Age group, years**						
19-29 (n=57)	47 (82.5%)	26 (45.6%)	15 (26.3%)	50 (87.7%)	17 (29.8%)	15 (26.3%)
30-39 (n=59)	52 (88.1%)	38 (64.4%)	25 (42.4%)	54 (91.5%)	13 (22.0%)	20 (33.9%)
40-49 (n=68)	59 (86.8%)	32 (47.1%)	25 (36.8%)	60 (88.2%)	12 (17.6%)	23 (33.8%)
50-59 (n=70)	53 (75.7%)	38 (54.3%)	36 (51.4%)	56 (80.0%)	18 (25.7%)	24 (34.3%)
≥60 (n=63)	51 (81.0%)	37 (58.7%)	27 (42.9%)	55 (87.3%)	19 (30.2%)	25 (39.7%)
P-value	0.341	0.194	0.065	0.387	0.425	0.661
**Symptom onset time**						
<4 weeks (n=31)	25 (80.7%)	19 (61.3%)	13 (41.9%)	27 (87.1%)	10 (32.3%)	7 (22.6%)
≥4 to <12 weeks (n=66)	54 (81.8%)	41 (62.1%)	31 (47.0%)	59 (89.4%)	19 (28.8%)	27 (20.8%)
≥12 weeks to <12 months (n=58)	48 (82.8%)	26 (44.8%)	18 (31.0%)	52 (89.7%)	9 (15.5%)	15 (25.9%)
≥12 months (n=162)	135 (83.3%)	85 (52.5%)	66 (40.7%)	137 (84.6%)	41 (25.3%)	58 (35.8%)
P-value	0.982	0.210	0.343	0.682	0.243	0.160
**Severity**						
Severe (n=113)	99 (87.6%)	75 (66.4%)	59 (52.2%)	99 (87.6%)	44 (38.9%)	54 (47.8%)
Moderate to severe (n=105)	89 (84.8%)	58 (55.2%)	41 (39.0%)	97 (92.4%)	19 (18.1%)	27 (25.7%)
Mild to moderate (n=99)	74 (74.7%)	38 (38.4%)	28 (28.3%)	79 (79.8%)	16 (16.2%)	26 (26.3%)
P-value	0.037	<0.001	0.002	0.028	<0.001	<0.001

All percentage values were calculated with the N in each row as the denominator.
