# Supplementary material for: Are Primary Outcomes Really Primary? An Exploratory Cross-Sectional Nationwide Web-Based Survey Study for Outcomes Reflecting Real Symptoms and Needs of Patients with Lumbar Disc Herniation
Source: Healthcare (Basel). 2023 Sep 21;11(18):2598. doi: 10.3390/healthcare11182598 (PMC10531434; doi:10.3390/healthcare11182598)
Supplement: Supplementary file 1 [file healthcare-11-02598-s001.zip › S2_Table.pdf]

Table S2. Types of disability due to lumbar disc herniation according to sex and age

	Total	Sex			Age (years)					
		Male	Female	*P*-value	19-29	30-39	40-49	50-59	≥60	*P*-value
Personal care	176 (35.2)	82 (32.4)	94 (38.1)	0.219	28 (28.0)	38 (38.0)	32 (32.0)	46 (46.0)	32 (32.0)	0.071
Lifting	318 (63.6)	159 (62.8)	159 (64.4)	0.794	50 (50.0)	66 (66.0)	65 (65.0)	69 (69.0)	68 (68.0)	0.034
Walking	215 (43.0)	93 (36.8)	122 (49.4)	0.006	37 (37.0)	34 (34.0)	39 (39.0)	46 (46.0)	59 (59.0)	0.003
Sitting	323 (64.6)	148 (58.5)	175 (70.9)	0.005	74 (74.0)	63 (63.0)	59 (59.0)	66 (66.0)	61 (61.0)	0.199
Standing	226 (45.2)	98 (38.7)	128 (51.8)	0.004	41 (41.0)	46 (46.0)	46 (46.0)	47 (47.0)	46 (46.0)	0.922
Sleeping	204 (40.8)	86 (34.0)	118 (47.8)	0.002	43 (43.0)	45 (45.0)	36 (36.0)	45 (45.0)	35 (35.0)	0.405
Traveling	210 (42.0)	115 (45.5)	95 (38.5)	0.135	32 (32.0)	37 (37.0)	54 (54.0)	45 (45.0)	42 (42.0)	0.022
Social life	119 (23.8)	60 (23.7)	59 (23.9)	1.000	15 (15.0)	19 (19.0)	24 (24.0)	35 (35.0)	26 (26.0)	0.013
Psychological	92 (18.4)	44 (17.4)	48 (19.4)	0.636	7 (7.0)	12 (12.0)	18 (18.0)	24 (24.0)	31 (31.0)	<0.001
Productivity loss	146 (29.2)	89 (35.2)	57 (23.1)	0.004	18 (18.0)	33 (33.0)	29 (29.0)	40 (40.0)	26 (26.0)	0.012
Gastro-internal	62 (12.4)	24 (9.5)	38 (15.4)	0.062	8 (8.0)	6 (6.0)	8 (8.0)	18 (18.0)	22 (22.0)	0.001
Others	9 (1.8)	3 (1.2)	6 (2.4)	0.334	4 (4.0)	1 (1.0)	2 (2.0)	1 (1.0)	1 (1.0)	0.572
