# Supplementary material for: Are Primary Outcomes Really Primary? An Exploratory Cross-Sectional Nationwide Web-Based Survey Study for Outcomes Reflecting Real Symptoms and Needs of Patients with Lumbar Disc Herniation
Source: Healthcare (Basel). 2023 Sep 21;11(18):2598. doi: 10.3390/healthcare11182598 (PMC10531434; doi:10.3390/healthcare11182598)
Supplement: Supplementary file 1 [file healthcare-11-02598-s001.zip › S3_Table.pdf]

Table S3. Types of disability due to lumbar disc herniation according to sex and age. (For patients diagnosed on MRI)

	Total	Sex			Age (years)					
		Male	Female	*P*-value	19-29	30-39	40-49	50-59	≥60	*P*-value
Personal care	116 (36.6%)	58 (33.7%)	58 (40.0%)	0.299	16 (28.1%)	20 (33.9%)	25 (36.8%)	32 (45.7%)	23 (36.5%)	0.345
Lifting	212 (66.9%)	113 (65.7%)	99 (68.3%)	0.714	30 (52.6%)	41 (69.5%)	46 (67.6%)	50 (71.4%)	45 (71.4%)	0.155
Walking	140 (44.2%)	67 (39.0%)	73 (50.3%)	0.055	**19 (33.3%)**	**18 (30.5%)**	**27 (39.7%)**	**34 (48.6%)**	**42 (66.7%)**	**<0.001**
Sitting	210 (66.2%)	**102 (59.3%)**	**108 (74.5%)**	**0.006**	42 (73.7%)	39 (66.1%)	42 (61.8%)	47 (67.1%)	40 (63.5%)	0.688
Standing	150 (47.3%)	**71 (41.3%)**	**79 (54.5%)**	**0.026**	21 (36.8%)	28 (47.5%)	34 (50.0%)	34 (48.6%)	33 (52.4%)	0.494
Sleeping	137 (43.2%)	**61 (35.5%)**	**76 (52.4%)**	**0.003**	26 (45.6%)	30 (50.8%)	23 (33.8%)	34 (48.6%)	24 (38.1%)	0.242
Traveling	147 (46.4%)	85 (49.4%)	62 (42.8%)	0.284	**18 (31.6%)**	**23 (39.0%)**	**44 (64.7%)**	**31 (44.3%)**	**31 (49.2%)**	**0.003**
Social life	89 (28.1%)	46 (26.7%)	43 (29.7%)	0.653	13 (22.8%)	14 (23.7%)	19 (27.9%)	24 (34.3%)	19 (30.2%)	0.59
Psychological	70 (22.1%)	34 (19.8%)	36 (24.8%)	0.344	**6 (10.5%)**	**9 (15.3%)**	**15 (22.1%)**	**20 (28.6%)**	**20 (31.7%)**	**0.025**
Productivity loss	110 (34.7%)	**70 (40.7%)**	**40 (27.6%)**	**0.02**	15 (26.3%)	22 (37.3%)	23 (33.8%)	29 (41.4%)	21 (33.3%)	0.491
Gastro-internal	48 (15.1%)	**16 (9.3%)**	**32 (22.1%)**	**0.003**	**5 (8.8%)**	**4 (6.8%)**	**6 (8.8%)**	**15 (21.4%)**	**18 (28.6%)**	**0.001**
Others	5 (1.6%)	3 (1.7%)	2 (1.4%)	**1.000**	**4 (7.0%)**	**0 (0.0%)**	**0 (0.0%)**	**0 (0.0%)**	**1 (1.6%)**	**0.003**
