# Supplementary material for: Are Primary Outcomes Really Primary? An Exploratory Cross-Sectional Nationwide Web-Based Survey Study for Outcomes Reflecting Real Symptoms and Needs of Patients with Lumbar Disc Herniation
Source: Healthcare (Basel). 2023 Sep 21;11(18):2598. doi: 10.3390/healthcare11182598 (PMC10531434; doi:10.3390/healthcare11182598)
Supplement: Supplementary file 1 [file healthcare-11-02598-s001.zip › S4_Table.pdf]

Table S4. Numeric rating scale scores for each symptom by respondent characteristics

	Back pain	Pelvic pain	Radiating leg pain	Numbness in the leg	Weakness in the leg	Tingling in the leg
Total	5.7 ± 2.2	5.6 ± 2.4	5.6 ± 2.5	5.0 ± 2.5	5.1 ± 2.5	4.7 ± 2.7
Sex						
Male	5.5 ± 2.3	5.6 ± 2.4	5.6 ± 2.3	4.9 ± 2.5	5.2 ± 2.3	4.7 ± 2.5
Female	5.8 ± 2.1	5.5 ± 2.4	5.6 ± 2.6	5.1 ± 2.4	5.0 ± 2.8	4.8 ± 2.9
P-value	0.111	0.808	0.901	0.288	0.650	0.808
Age, years						
19-29	5.2 ± 2.2	5.4 ± 2.4	5.2 ± 2.7	4.6 ± 2.4	4.4 ± 2.8	4.1 ± 2.9
30-39	5.4 ± 2.3	5.3 ± 2.5	5.6 ± 2.3	4.5 ± 2.6	4.2 ± 2.9	3.9 ± 2.6
40-49	5.3 ± 2.2	5.1 ± 2.4	4.5 ± 2.6	4.8 ± 2.2	5.1 ± 2.1	4.9 ± 2.5
50-59	6.2 ± 2.1	5.9 ± 2.3	6.5 ± 2.2	5.5 ± 2.6	5.6 ± 1.9	5.3 ± 2.5
≥60	6.2 ± 2.3	6.1 ± 2.4	5.8 ± 2.1	5.5 ± 2.5	6.1 ± 2.4	5.0 ± 2.8
P-value	0.002	0.185	0.008	0.008	0.057	0.197
Symptom onset time						
<4 weeks	5.6 ± 2.1	5.6 ± 2.3	5.7 ± 2.3	5.0 ± 2.4	6.1 ± 2.5	4.1 ± 2.5
≥4 weeks to <12 weeks	5.9 ± 2.1	5.9 ± 2.3	5.8 ± 2.2	5.0 ± 2.4	4.6 ± 2.3	4.8 ± 2.7
≥12 weeks to <12 months	5.3 ± 2.3	5.1 ± 2.6	5.4 ± 2.5	4.9 ± 2.4	5.3 ± 1.9	5.6 ± 2.3
≥12 months	5.7 ± 2.3	5.5 ± 2.5	5.6 ± 2.6	5.0 ± 2.6	5.2 ± 2.8	4.5 ± 2.8
P-value	0.360	0.435	0.865	0.981	0.316	0.282
Severity						
Severe	7.8 ± 1.5	7.7 ± 1.5	5.6 ± 2.5	7.2 ± 2.0	6.5 ± 2.4	6.3 ± 2.5
Moderate to severe	5.9 ± 1.2	5.6 ± 1.4	5.7 ± 1.4	5.3 ± 1.6	5.1 ± 1.5	4.6 ± 2.1
Mild to moderate	3.3 ± 1.4	2.8 ± 1.6	2.7 ± 1.4	2.7 ± 1.5	2.8 ± 1.6	2.4 ± 1.4
P-value	<0.001	<0.001	<0.001	<0.001	<0.001	<0.001

Symptom severity is defined according to the NRS score as follows: ≥8, severe; 6–7, moderate to severe; ≤5, mild to moderate.
