# Supplementary material for: Are Primary Outcomes Really Primary? An Exploratory Cross-Sectional Nationwide Web-Based Survey Study for Outcomes Reflecting Real Symptoms and Needs of Patients with Lumbar Disc Herniation
Source: Healthcare (Basel). 2023 Sep 21;11(18):2598. doi: 10.3390/healthcare11182598 (PMC10531434; doi:10.3390/healthcare11182598)
Supplement: Supplementary file 1 [file healthcare-11-02598-s001.zip › S5_Table.pdf]

Table S5. Severity of disability according to sex and age

	Total	Sex		Age (years)				
		Male	Female	19-29	30-39	40-49	50-59	60≤
**Personal care (N=176)**		82	94	28	38	32	46	32
1	100 (56.8)	37 (45.1)	63 (67.0)	21 (75.0)	26 (68.4)	15 (46.9)	20 (43.5)	18 (56.2)
2	53 (30.1)	33 (40.2)	20 (21.3)	7 (25.0)	8 (21.1)	10 (31.2)	18 (39.1)	10 (31.2)
3	14 (8.0)	9 (11.0)	5 (5.3)	0 (0.0)	4 (10.5)	5 (15.6)	4 (8.7)	1 (3.1)
4	1 (0.6)	0 (0.0)	1 (1.1)	0 (0.0)	0 (0.0)	0 (0.0)	1 (2.2)	0 (0.0)
5	8 (4.5)	3 (3.7)	5 (5.3)	0 (0.0)	0 (0.0)	2 (6.2)	3 (6.5)	3 (9.4)
P-value		0.012		0.159				
**Lifting (N=318)**		159	159	50	66	65	69	68
1	128 (40.3)	74 (46.5)	54 (34.0)	33 (66.0)	34 (51.5)	25 (38.5)	22 (31.9)	14 (20.6)
2	61 (19.2)	33 (20.8)	28 (17.6)	8 (16.0)	11 (16.7)	15 (23.1)	16 (23.2)	11 (16.2)
3	80 (25.2)	29 (18.2)	51 (32.1)	6 (12.0)	13 (19.7)	16 (24.6)	19 (27.5)	26 (38.2)
4	39 (12.3)	19 (11.9)	20 (12.6)	3 (6.0)	7 (10.6)	6 (9.2)	9 (13.0)	14 (20.6)
5	10 (3.1)	4 (2.5)	6 (3.8)	0 (0.0)	1 (1.5)	3 (4.6)	3 (4.3)	3 (4.4)
P-value		0.040		0.001				
**Walking (N=215)**		93	122	37	34	39	46	59
1	167 (77.7)	73 (78.5)	94 (77.0)	30 (81.1)	29 (85.3)	31 (79.5)	29 (63.0)	48 (81.4)
2	19 (8.8)	9 (9.7)	10 (8.2)	5 (13.5)	0 (0.0)	4 (10.3)	5 (10.9)	5 (8.5)
3	19 (8.8)	7 (7.5)	12 (9.8)	1 (2.7)	4 (11.8)	2 (5.1)	8 (17.4)	4 (6.8)
4	1 (0.5)	0 (0.0)	1 (0.8)	0 (0.0)	1 (2.9)	0 (0.0)	0 (0.0)	0 (0.0)
5	9 (4.2)	4 (4.3)	5 (4.1)	1 (2.7)	0 (0.0)	2 (5.1)	4 (8.7)	2 (3.4)
P-value		0.966		0.131				
**Sitting (N=323)**		148	175	74	63	59	66	61
1	136 (42.1)	60 (40.5)	76 (43.4)	45 (60.8)	32 (50.8)	20 (33.9)	17 (25.8)	22 (36.1)
2	117 (36.2)	56 (37.8)	61 (34.9)	18 (24.3)	22 (34.9)	30 (50.8)	25 (37.9)	22 (36.1)

3	52 (16.1)	24 (16.2)	28 (16.0)	9 (12.2)	7 (11.1)	5 (8.5)	19 (28.8)	12 (19.7)
4	13 (4.0)	5 (3.4)	8 (4.6)	1 (1.4)	2 (3.2)	3 (5.1)	4 (6.1)	3 (4.9)
5	5 (1.5)	3 (2.0)	2 (1.1)	1 (1.4)	0 (0.0)	1 (1.7)	1 (1.5)	2 (3.3)
P-value		0.906		0.004				
**Standing (N=226)**		98	128	41	46	46	47	46
1	129 (57.1)	53 (54.1)	76 (59.4)	30 (73.2)	29 (63.0)	22 (47.8)	22 (46.8)	26 (56.5)
2	43 (19.0)	25 (25.5)	18 (14.1)	7 (17.1)	9 (19.6)	13 (28.3)	8 (17.0)	6 (13.0)
3	38 (16.8)	13 (13.3)	25 (19.5)	3 (7.3)	7 (15.2)	8 (17.4)	11 (23.4)	9 (19.6)
4	14 (6.2)	7 (7.1)	7 (5.5)	1 (2.4)	1 (2.2)	2 (4.3)	6 (12.8)	4 (8.7)
5	2 (0.9)	0 (0.0)	2 (1.6)	0 (0.0)	0 (0.0)	1 (2.2)	0 (0.0)	1 (2.2)
P-value		0.126		0.228				
**Sleeping (N=204)**		86	118	43	45	36	45	35
1	115 (56.4)	48 (55.8)	67 (56.8)	31 (72.1)	33 (73.3)	20 (55.6)	18 (40.0)	13 (37.1)
2	37 (18.1)	13 (15.1)	24 (20.3)	7 (16.3)	5 (11.1)	8 (22.2)	7 (15.6)	10 (28.6)
3	32 (15.7)	16 (18.6)	16 (13.6)	5 (11.6)	4 (8.9)	4 (11.1)	11 (24.4)	8 (22.9)
4	19 (9.3)	9 (10.5)	10 (8.5)	0 (0.0)	3 (6.7)	3 (8.3)	9 (20.0)	4 (11.4)
5	1 (0.5)	0 (0.0)	1 (0.8)	0 (0.0)	0 (0.0)	1 (2.8)	0 (0.0)	0 (0.0)
P-value		0.681		0.005				
**Traveling (N=210)**		115	95	32	37	54	45	42
1	88 (41.9)	56 (48.7)	32 (33.7)	14 (43.8)	17 (45.9)	26 (48.1)	13 (28.9)	18 (42.9)
2	73 (34.8)	39 (33.9)	34 (35.8)	14 (43.8)	13 (35.1)	18 (33.3)	15 (33.3)	13 (31.0)
3	37 (17.6)	16 (13.9)	21 (22.1)	3 (9.4)	6 (16.2)	6 (11.1)	12 (26.7)	10 (23.8)
4	2 (1.0)	1 (0.9)	1 (1.1)	1 (3.1)	0 (0.0)	0 (0.0)	1 (2.2)	0 (0.0)
5	10 (4.8)	3 (2.6)	7 (7.4)	0 (0.0)	1 (2.7)	4 (7.4)	4 (8.9)	1 (2.4)
P-value		0.091		0.353				
**Social life (N=119)**		60	59	15	19	24	35	26
1	41 (34.5)	18 (30.0)	23 (39.0)	8 (53.3)	9 (47.4)	6 (25.0)	10 (28.6)	8 (30.8)

2	48 (40.3)	28 (46.7)	20 (33.9)	2 (13.3)	7 (36.8)	14 (58.3)	13 (37.1)	12 (46.2)
3	20 (16.8)	11 (18.3)	9 (15.3)	3 (20.0)	3 (15.8)	0 (0.0)	8 (22.9)	6 (23.1)
4	5 (4.2)	2 (3.3)	3 (5.1)	1 (6.7)	0 (0.0)	2 (8.3)	2 (5.7)	0 (0.0)
5	5 (4.2)	1 (1.7)	4 (6.8)	1 (6.7)	0 (0.0)	2 (8.3)	2 (5.7)	0 (0.0)
P-value		0.396		0.186				
**Anxiety/depression (N=92)**		44	48	7	12	18	24	31
1	3 (3.3)	0 (0.0)	3 (6.2)	1 (14.3)	0 (0.0)	0 (0.0)	2 (8.3)	0 (0.0)
2	43 (46.7)	22 (50.0)	21 (43.8)	3 (42.9)	5 (41.7)	9 (50.0)	10 (41.7)	16 (51.6)
3	36 (39.1)	18 (40.9)	18 (37.5)	1 (14.3)	5 (41.7)	7 (38.9)	11 (45.8)	12 (38.7)
4	10 (10.9)	4 (9.1)	6 (12.5)	2 (28.6)	2 (16.7)	2 (11.1)	1 (4.2)	3 (9.7)
5	-	-	-	-	-	-	-	-
P-value		0.441		0.473				
**Productivity loss (N=146)**		89	57	18	33	29	40	26
1	64 (43.8)	38 (42.7)	26 (45.6)	7 (38.9)	15 (45.5)	18 (62.1)	18 (45.0)	6 (23.1)
2	39 (26.7)	26 (29.2)	13 (22.8)	6 (33.3)	10 (30.3)	5 (17.2)	11 (27.5)	7 (26.9)
3	22 (15.1)	13 (14.6)	9 (15.8)	5 (27.8)	5 (15.2)	3 (10.3)	3 (7.5)	6 (23.1)
4	9 (6.2)	5 (5.6)	4 (7.0)	0 (0.0)	1 (3.0)	1 (3.4)	5 (12.5)	2 (7.7)
5	12 (8.2)	7 (7.9)	5 (8.8)	0 (0.0)	2 (6.1)	2 (6.9)	3 (7.5)	5 (19.2)
P-value		0.935		0.161				
**Gastrointestinal problems (N=62)**		24	38	8	6	8	18	22
1	8 (12.9)	2 (8.3)	6 (15.8)	1 (12.5)	0 (0.0)	1 (12.5)	2 (11.1)	4 (18.2)
2	24 (38.7)	9 (37.5)	15 (39.5)	3 (37.5)	3 (50.0)	5 (62.5)	7 (38.9)	6 (27.3)
3	22 (35.5)	11 (45.8)	11 (28.9)	2 (25.0)	3 (50.0)	1 (12.5)	6 (33.3)	10 (45.5)
4	8 (12.9)	2 (8.3)	6 (15.8)	2 (25.0)	0 (0.0)	1 (12.5)	3 (16.7)	2 (9.1)
5	-	-	-	-	-	-	-	-
P-value		0.541		0.819				

Differences according to age were analyzed using the chi-square test with the simulated *P*-value calculated using the Monte Carlo method.
