# Supplementary material for: Are Primary Outcomes Really Primary? An Exploratory Cross-Sectional Nationwide Web-Based Survey Study for Outcomes Reflecting Real Symptoms and Needs of Patients with Lumbar Disc Herniation
Source: Healthcare (Basel). 2023 Sep 21;11(18):2598. doi: 10.3390/healthcare11182598 (PMC10531434; doi:10.3390/healthcare11182598)
Supplement: Supplementary file 1 [file healthcare-11-02598-s001.zip › S6_Table.pdf]

Table S6. Priority symptoms for improvement by respondent characteristics. (For patients diagnosed on MRI)

	Back pain	Pelvic pain	Radiating leg pain	Numbness in the leg	Weakness in the leg	Tingling in the leg
	N (%)	N (%)	N (%)	N (%)	N (%)	N (%)
**Total (N=317)**	194 (61.2%)	98 (30.9%)	67 (21.1%)	144 (45.4%)	25 (7.9%)	34 (10.7%)
**Sex**						
Male (n=172)	105 (61.0%)	44 (25.6%)	36 (20.9%)	81 (47.1%)	16 (9.3%)	22 (12.8%)
Female (n=145)	89 (61.4%)	54 (37.2%)	31 (21.4%)	63 (43.4%)	9 (6.2%)	12 (8.3%)
P-value	1.000	0.034	1.000	0.592	0.418	0.266
**Age group, years**						
19-29 (n=57)	39 (68.4%)	14 (24.6%)	9 (15.8%)	24 (42.1%)	7 (12.3%)	3 (5.3%)
30-39 (n=59)	39 (66.1%)	18 (30.5%)	15 (25.4%)	25 (42.4%)	4 (6.8%)	7 (11.9%)
40-49 (n=68)	46 (67.6%)	20 (29.4%)	12 (17.6%)	34 (50.0%)	3 (4.4%)	10 (14.7%)
50-59 (n=70)	38 (54.3%)	20 (28.6%)	18 (25.7%)	33 (47.1%)	5 (7.1%)	10 (14.3%)
≥60 (n=63)	32 (50.8%)	26 (41.3%)	13 (20.6%)	28 (44.4%)	6 (9.5%)	4 (6.3%)
P-value	0.120	0.343	0.555	0.885	0.564	0.270
**Symptom onset time**						
<4 weeks (n=31)	18 (58.1%)	6 (19.4%)	7 (22.6%)	17 (54.8%)	3 (9.7%)	2 (6.5%)
≥4 weeks to <12 weeks (n=66)	42 (63.6%)	19 (28.8%)	17 (25.8%)	30 (45.5%)	7 (10.6%)	7 (10.6%)
≥12 weeks to <12 months (n=58)	37 (63.8%)	17 (29.3%)	8 (13.8%)	30 (51.7%)	2 (3.5%)	10 (17.2%)
≥12 months (n=162)	97 (59.9%)	56 (34.6%)	35 (21.6%)	67 (41.4%)	13 (8.0%)	15 (9.3%)
P-value	0.902	0.367	0.427	0.374	0.458	0.317
**Severity**						
Severe (n=113)	69 (61.1%)	43 (38.1%)	28 (24.8%)	46 (40.7%)	10 (8.8%)	16 (14.2%)
Moderate to severe (n=105)	68 (64.8%)	35 (33.3%)	22 (21.0%)	49 (46.7%)	6 (5.7%)	9 (8.6%)
Mild to moderate (n=99)	57 (57.6%)	20 (20.2%)	17 (17.2%)	49 (49.5%)	9 (9.1%)	9 (9.1%)
P-value	0.574	0.016	0.399	0.419	0.599	0.337

All percentage values were calculated with the n in each row as the denominator.

Symptom severity is defined according to the NRS score as follows: ≥8, severe; 6-7, moderate to severe; ≤5, mild to moderate.
