# Supplementary material for: Are Primary Outcomes Really Primary? An Exploratory Cross-Sectional Nationwide Web-Based Survey Study for Outcomes Reflecting Real Symptoms and Needs of Patients with Lumbar Disc Herniation
Source: Healthcare (Basel). 2023 Sep 21;11(18):2598. doi: 10.3390/healthcare11182598 (PMC10531434; doi:10.3390/healthcare11182598)
Supplement: Supplementary file 1 [file healthcare-11-02598-s001.zip › S8_Table.pdf]

Table S8. Priority disabilities for improvement

	Total	Sex			Age (years)					
		Male	Female	*P*-value	19-29	30-39	40-49	50-59	60≤	*P*-value
**Total (N=500)**		253	247		100	100	100	100	100	
Personal care	79 (15.8)	37 (14.6)	42 (17.0)	0.544	12 (12.0)	16 (16.0)	14 (14.0)	22 (22.0)	15 (15.0)	0.371
Lifting	103 (20.6)	57 (22.5)	46 (18.6)	0.332	15 (15.0)	34 (34.0)	23 (23.0)	14 (14.0)	17 (17.0)	0.002
Walking	96 (19.2)	39 (15.4)	57 (23.1)	**0.039**	11 (11.0)	16 (16.0)	22 (22.0)	17 (17.0)	30 (30.0)	0.010
Sitting	184 (36.8)	78 (30.8)	106 (42.9)	**0.007**	53 (53.0)	36 (36.0)	37 (37.0)	28 (28.0)	30 (30.0)	0.002
Standing	82 (16.4)	34 (13.4)	48 (19.4)	**0.091**	24 (24.0)	12 (12.0)	12 (12.0)	17 (17.0)	17 (17.0)	0.131
Sleeping	106 (21.2)	47 (18.6)	59 (23.9)	0.179	28 (28.0)	21 (21.0)	21 (21.0)	18 (18.0)	18 (18.0)	0.406
Traveling	72 (14.4)	50 (19.8)	22 (8.9)	**0.001**	8 (8.0)	11 (11.0)	21 (21.0)	17 (17.0)	15 (15.0)	0.079
Social life	29 (5.8)	18 (7.1)	11 (4.5)	0.279	4 (4.0)	4 (4.0)	8 (8.0)	7 (7.0)	6 (6.0)	0.673
Anxiety/Depression	27 (5.4)	16 (6.3)	11 (4.5)	0.467	3 (3.0)	6 (6.0)	5 (5.0)	7 (7.0)	6 (6.0)	0.772
Productivity loss	67 (13.4)	50 (19.8)	17 (6.9)	<0.001	8 (8.0)	19 (19.0)	10 (10.0)	19 (19.0)	11 (11.0)	0.052
Gastrointestinal	19 (3.8)	10 (4.0)	9 (3.6)	1.000	3 (3.0)	1 (1.0)	1 (1.0)	6 (6.0)	**8 (8.0)**	0.03
Others	8 (1.6)	3 (1.2)	5 (2.0)	0.500	4 (4.0)	0 (0.0)	2 (2.0)	1 (1.0)	1 (1.0)	0.293

All values are represented with frequency and percentage.
