# Supplementary material for: Are Primary Outcomes Really Primary? An Exploratory Cross-Sectional Nationwide Web-Based Survey Study for Outcomes Reflecting Real Symptoms and Needs of Patients with Lumbar Disc Herniation
Source: Healthcare (Basel). 2023 Sep 21;11(18):2598. doi: 10.3390/healthcare11182598 (PMC10531434; doi:10.3390/healthcare11182598)
Supplement: Supplementary file 1 [file healthcare-11-02598-s001.zip › S9_Table.pdf]

Table S9. Priority disabilities for improvement. (For patients diagnosed on MRI)

| | Total | Sex | | | Age (years) | | | | | |
|---|---|---|---|---|---|---|---|---|---|---|
| | | Male | Female | *P*-value | 19-29 | 30-39 | 40-49 | 50-59 | 60≤ | *P*-value |
| **Total (N=317)** | | 172 | 145 | | 57 | 59 | 68 | 70 | 63 | |
| Personal care | 48 (15.1%) | 23 (13.4%) | 25 (17.2%) | 0.424 | 7 (12.3%) | 7 (11.9%) | 10 (14.7%) | 15 (21.4%) | 9 (14.3%) | 0.549 |
| Lifting | 68 (21.5%) | 38 (22.1%) | 30 (20.7%) | 0.868 | 8 (14.0%) | **20 (33.9%)** | 18 (26.5%) | 13 (18.6%) | 9 (14.3%) | **0.032** |
| Walking | 55 (17.4%) | **22 (12.8%)** | **33 (22.8%)** | **0.029** | 3 (5.3%) | 6 (10.2%) | 13 (19.1%) | 12 (17.1%) | **21 (33.3%)** | **0.001** |
| Sitting | 120 (37.9%) | **55 (32.0%)** | **65 (44.8%)** | **0.025** | 28 (49.1%) | 26 (44.1%) | 26 (38.2%) | 21 (30.0%) | 19 (30.2%) | 0.113 |
| Standing | 44 (13.9%) | 22 (12.8%) | 22 (15.2%) | 0.654 | 11 (19.3%) | 5 (8.5%) | 8 (11.8%) | 11 (15.7%) | 9 (14.3%) | 0.509 |
| Sleeping | 69 (21.8%) | 32 (18.6%) | 37 (25.5%) | 0.177 | 19 (33.3%) | 14 (23.7%) | 10 (14.7%) | 13 (18.6%) | 13 (20.6%) | 0.132 |
| Traveling | 53 (16.7%) | **37 (21.5%)** | **16 (11.0%)** | **0.019** | 4 (7.0%) | 9 (15.3%) | **18 (26.5%)** | 12 (17.1%) | 10 (15.9%) | 0.071 |
| Social life | 21 (6.6%) | 13 (7.6%) | 8 (5.5%) | 0.616 | 3 (5.3%) | 4 (6.8%) | 5 (7.4%) | 5 (7.1%) | 4 (6.3%) | 0.994 |
| Anxiety/Depression | 21 (6.6%) | 13 (7.6%) | 8 (5.5%) | 0.616 | 2 (3.5%) | 5 (8.5%) | 4 (5.9%) | 6 (8.6%) | 4 (6.3%) | 0.8 |
| Productivity loss | 46 (14.5%) | **37 (21.5%)** | **9 (6.2%)** | **<0.001** | 6 (10.5%) | 10 (16.9%) | 7 (10.3%) | 14 (20.0%) | 9 (14.3%) | 0.45 |
| Gastrointestinal | 15 (4.7%) | 6 (3.5%) | 9 (6.2%) | 0.384 | 2 (3.5%) | 1 (1.7%) | 1 (1.5%) | 5 (7.1%) | 6 (9.5%) | 0.15 |
| Others | 5 (1.6%) | 3 (1.7%) | 2 (1.4%) | 1 | 4 (7.0%) | 0 (0.0%) | 0 (0.0%) | 0 (0.0%) | 1 (1.6%) | 0.003 |

All values are represented with frequency and percentage.
