# Supplementary material for: Are Primary Outcomes Really Primary? An Exploratory Cross-Sectional Nationwide Web-Based Survey Study for Outcomes Reflecting Real Symptoms and Needs of Patients with Lumbar Disc Herniation
Source: Healthcare (Basel). 2023 Sep 21;11(18):2598. doi: 10.3390/healthcare11182598 (PMC10531434; doi:10.3390/healthcare11182598)
Supplement: Supplementary file 1 [file healthcare-11-02598-s001.zip › Table S14.pdf]

Table S14. Unmet medical needs and desire for treatment

	Total	Sex			Age (years)					
		Male	Female	*P*-value	19-29	30-39	40-49	50-59	60≤	*P*-value
**Sufficient care**										
Yes	268 (53.6)	143 (56.5)	125 (50.6)	0.185	50 (50.0)	52 (52.0)	60 (60.0)	52 (52.0)	54 (54.0)	0.67
No	232 (46.4)	110 (43.5)	122 (49.4)		50 (50.0)	48 (48.0)	40 (40.0)	48 (48.0)	46 (46.0)	
**Reasons for unmet medical needs (N=232)**		110	122		50	48	40	48	46	
Unsatisfactory effect	112 (48.3)	55 (50.0)	57 (46.7)	0.713	15 (30.0)	21 (43.8)	20 (50.0)	27 (56.2)	29 (63.0)	0.02
Cost	152 (65.5)	71 (64.5)	81 (66.4)	0.875	35 (70.0)	37 (77.1)	25 (62.5)	30 (62.5)	25 (54.3)	0.19
Time	158 (68.1)	74 (67.3)	84 (68.9)	0.907	34 (68.0)	36 (75.0)	33 (82.5)	35 (72.9)	20 (43.5)	0.001
Accessibility	60 (25.9)	31 (28.2)	29 (23.8)	0.538	16 (32.0)	9 (18.8)	11 (27.5)	16 (33.3)	8 (17.4)	0.25
Physician does not take the symptom seriously	36 (15.5)	22 (20.0)	14 (11.5)	0.108	6 (12.0)	5 (10.4)	6 (15.0)	7 (14.6)	12 (26.1)	0.25
Others	1 (0.4)	1 (0.9)	0 (0.0)	0.474	0 (0.0)	0 (0.0)	0 (0.0)	0 (0.0)	1 (2.2)	0.37
**Desire for treatment**										
No treatment is needed when symptoms improve	194 (38.8)	79 (31.2)	115 (46.6)	0.001	53 (53.0)	42 (42.0)	32 (32.0)	36 (36.0)	31 (31.0)	0.01
Treatment should be continued even after improvement	306 (61.2)	174 (68.8)	132 (53.4)		47 (47.0)	58 (58.0)	68 (68.0)	64 (64.0)	69 (69.0)	
**Desire for treatment (Frequency)**										
4 times or more per week	43 (8.6)	27 (10.7)	16 (6.5)	0.411	13 (13.0)	9 (9.0)	4 (4.0)	10 (10.0)	7 (7.0)	0.06
2–3 times per week	232 (46.4)	114 (45.1)	118 (47.8)		46 (46.0)	39 (39.0)	52 (52.0)	50 (50.0)	45 (45.0)	
Once a week	157 (31.4)	79 (31.2)	78 (31.6)		27 (27.0)	32 (32.0)	34 (34.0)	35 (35.0)	29 (29.0)	
Two times or less per month	68 (13.6)	33 (13.0)	35 (14.2)		14 (14.0)	20 (20.0)	10 (10.0)	5 (5.0)	19 (19.0)	
**Desire for treatment (Duration per session)**										
Less than 1 hour	391 (78.2)	193 (76.3)	198 (80.2)	0.153	82 (82.0)	73 (73.0)	76 (76.0)	82 (82.0)	78 (78.0)	0.15

Between 1 and 2 hours	102 (20.4)	54 (21.3)	48 (19.4)		17 (17.0)	23 (23.0)	22 (22.0)	18 (18.0)	22 (22.0)	
2 hours or longer	7 (1.4)	6 (2.4)	1 (0.4)		1 (1.0)	4 (4.0)	2 (2.0)	0 (0.0)	0 (0.0)	
